# METTL14-mediated m^6^A methylation orchestrates early NK cell development by maintaining metabolic fitness

**DOI:** 10.1038/s41419-026-09069-2

**Published:** 2026-07-28

**Authors:** Huan Ma, Zhenzhen Tu, Surong Deng, Yirong Sun, Shuxin Wang, Hongxia Wang, Chen Cheng, Xiang Li, Zhongjun Dong, Shasha Chen

**Affiliations:** 1 The First Affiliated Hospital of Anhui Medical University and Institute of Clinical Immunology, Anhui Medical University, Hefei, China; 2 Department of Immunology, School of Basic Medical Sciences, Anhui Medical University, Hefei, China; 3 Department of Immunobiology, School of Life Sciences, Anhui Medical University, Hefei, China; 4 School of Basic Medicine and Institute for Immunology, Tsinghua University, Beijing, China; 5 Innovative Institute of Tumor Immunity and Medicine (ITIM), Hefei, China; 6 Anhui Province Key Laboratory of Tumor Immune Microenvironment and Immunotherapy, Hefei, China; 7 Inflammation and Immune Mediated Diseases Laboratory of Anhui Province, Anhui Medical University, Hefei, China

**Keywords:** Innate lymphoid cells, NK cells, Immune cell death

## Abstract

N6-methyladenosine (m^6^A) represents the most abundant mRNA modification, yet its role in natural killer (NK) cell development remains incompletely understood. Here we demonstrate that the m^6^A methyltransferase METTL14 plays an indispensable, stage-specific role in early NK cell development. *Mettl14* deficiency at the progenitor stage caused severe NK cell lymphopenia by disrupting the NK progenitor to immature NK cell transition. Paradoxically, residual *Mettl14*-null NK cells exhibited a hypermetabolic state characterized by mTORC1 hyperactivation and enhanced mitochondrial function, which drove both hyperproliferation and activation-induced cell death via p53 and apoptotic pathway activation. Despite developmental defects, these cells demonstrated superior capacity to control melanoma metastasis in vivo. Mechanistically, METTL14 fine-tuned IL-15 responsiveness likely by sustaining SOCS3 expression to restrain JAK-STAT5 signaling. Terminal deletion of *Mettl14* produced no phenotype, underscoring its specific requirement during early development. Our findings establish METTL14 as a crucial checkpoint coordinating transcriptional and metabolic programs to ensure NK cell homeostasis.

**Figure:**
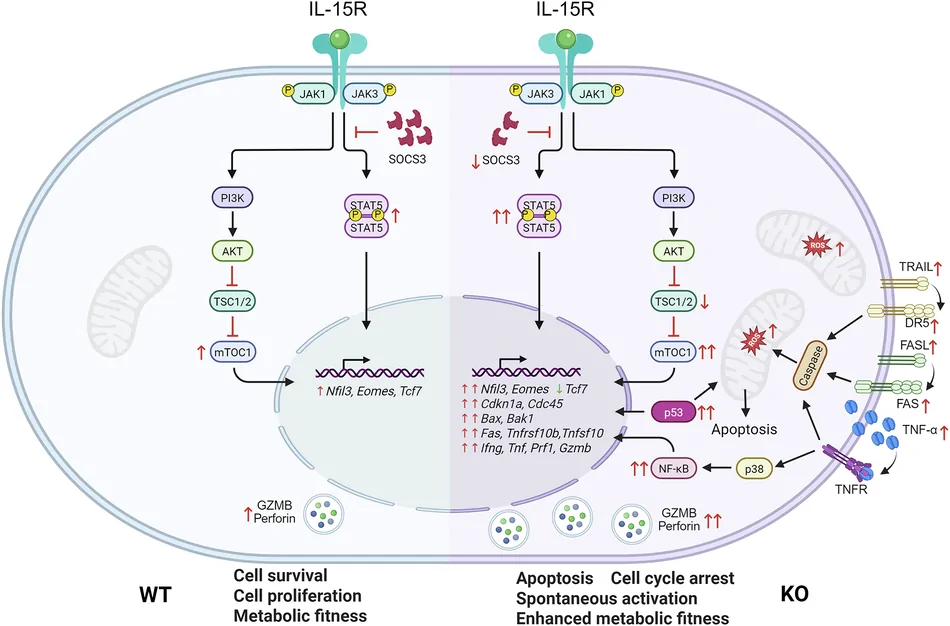

## Introduction

Natural killer (NK) cells are innate lymphocytes critical for the first-line defense against infected or malignant cells. They directly eliminate targets via cytotoxic mediators (granzyme B, perforin) or death receptors (FASL, TRAIL) and secrete potent antiviral/antitumor cytokines like IFN-γ and TNF-α [1–3].

NK cells develop from common lymphoid progenitors (CLPs) in the bone marrow, progressing through NK progenitor (NKP) stages marked by CD122 (IL-2/15Rβ) expression [4]. CD122 is pivotal for NK cell lineage commitment by enabling responsiveness to IL-15. IL-15/IL15R signaling induces key transcription factors (E4BP4, Eomes, ID2, T-bet, ETS1) essential for development, maturation, survival, and function through JAK-STAT5, PI3K-AKT-mTOR, and MAPK pathways [5–12]. While transcriptional regulation is well-characterized, post-transcriptional control of NK cell development and homeostasis remains poorly understood.

Resting NK cells constitutively transcribe effector genes (*Gzmb*, *Prf1*, *Ifng*) but exhibit minimal protein expression [13, 14], indicating stringent post-transcriptional restraint (e.g., mRNA stability/ translation control) to prevent autoreactivity and maintain a poised state until activation [15]. The mechanisms underlying this regulation are unclear.

N6-methyladenosine (m^6^A), the predominant mRNA modification, is dynamically regulated by writers, erasers, and readers. The METTL3/METTL14 complex is the primary writer, with METTL14 crucial for substrate recognition [16–18]. m^6^A influences mRNA splicing, translation, stability, and degradation [16], and its dysregulation disrupts hematopoiesis and immune cell development/function [19–30]. Studies using *Mettl3* (writer)-, *Ythdf2* (eraser)-, or *Fto* (eraser)-deficient mice implicate m^6^A in NK cell homeostasis/function [25, 31, 32]. However, stage-specific role of m^6^A modification, particularly within early NK cells, remain poorly defined.

In this study, we observed peak expression of m^6^A machinery components in NKPs, suggesting a key role in early ontogeny. To test this, we conditionally deleted *Mettl14* (a core methyltransferase component) in early NK cells using CD122-Cre. This study demonstrates that METTL14-mediated m^6^A methylation is essential for NK cell homeostasis and maturation. *Mettl14* deficiency caused severe lymphopenia and impaired maturation due to disrupted gene programs critical for the NKP-to-immature NK (iNK) transition. Residual *Mettl14*-deficient NK cells displayed a hyperactivated phenotype. These findings establish a vital role for m^6^A in maintaining metabolic fitness and regulating early NK cell developmental checkpoints.

## Results

### Dynamic changes in m6A-modified transcripts across NK cell development

**Fig. 1:**
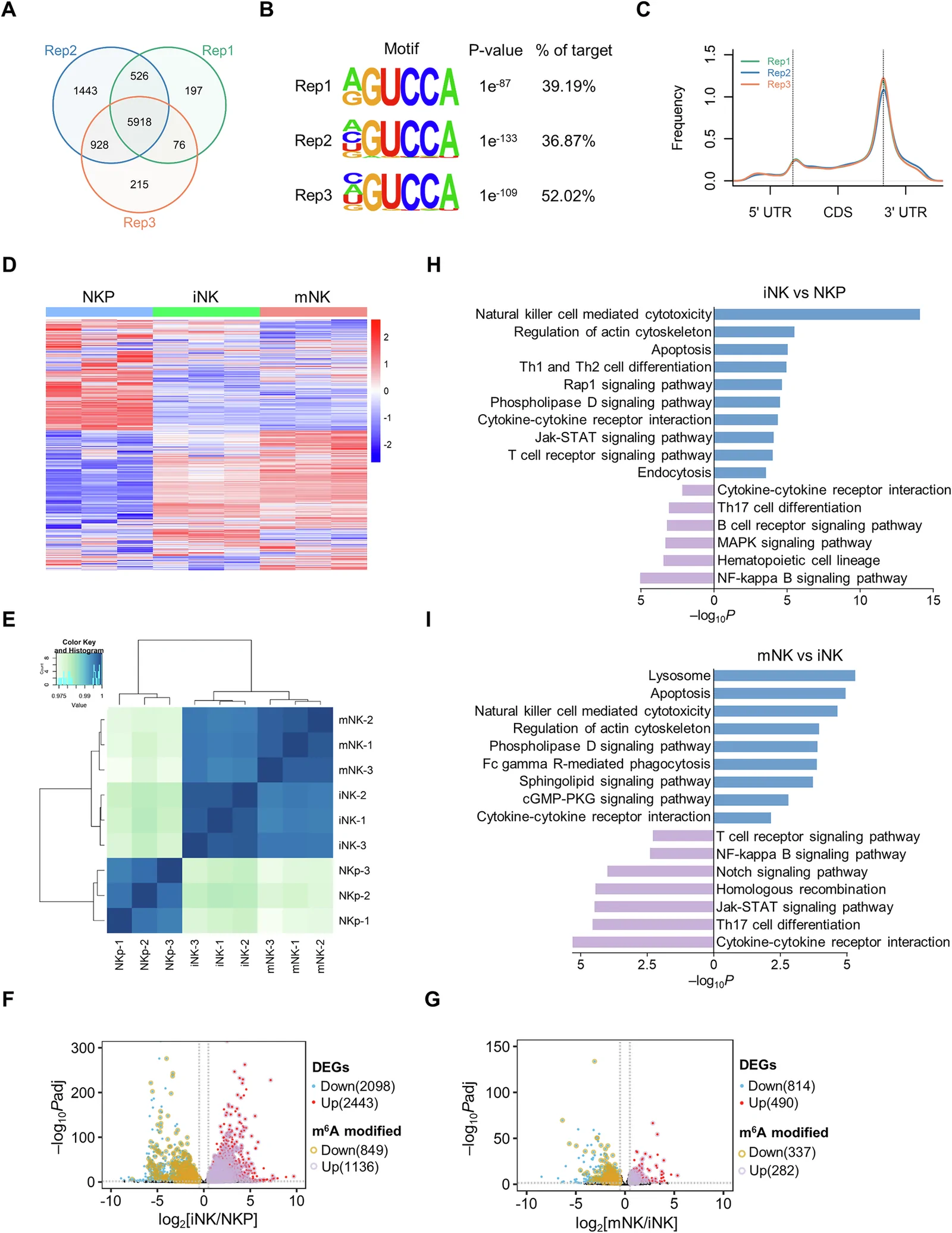
Dynamic changes in m^6^A-modified transcripts across NK cell development. **A** Venn diagram showing overlapping m⁶A-modified transcripts identified by meRIP-seq in three biological replicates of WT NK cells (CD3⁻NK1.1⁺). **B** Summary of top consensus motifs within m⁶A peaks, their p-values, and occurrence frequencies in three WT NK cell replicates. **C** Metagene profiles illustrating transcriptome-wide m⁶A peak distribution across three WT NK cell replicates. **D** Heatmap of m⁶A-modified gene expression in NKP (CD3⁻CD122⁺NK1.1⁻CD11b⁻), iNK (CD3⁻CD122⁺NK1.1⁺CD11b⁻), and mNK (CD3⁻CD122⁺NK1.1⁺ CD11b⁺) cell subsets sorted from WT splenocytes. **E** Heatmap of pairwise Pearson correlation coefficients for normalized gene expression across WT NKP, iNK, and mNK cells. Volcano plots of differentially expressed transcripts (adjusted *P* ≤ 0.05; log_2_ fold-change ≥0.5) in iNK versus NKP (**F**) and mNK versus iNK (**G**). m^6^A-modified genes are marked with circles. KEGG pathway enrichment of differentially expressed m⁶A-modified genes in iNK versus NKP (**H**) and mNK versus iNK (**I**).

To investigate the potential roles of m^6^A methylation in NK cell biology, we mapped the global m^6^A landscape in murine NK cells using Methylated RNA Immunoprecipitation Sequencing (MeRIP-seq). Quality control (QC) analysis using deepTools indicated high concordance among biological replicates (Supplementary Fig. S1A, B). Picard-based QC analysis of the RNA-Seq coverage profile across transcript positions showed high reproducibility among biological replicates, with no obvious degradation or bias, indicating optimal library construction (Supplementary Fig. S1C). The genomic region distribution further validated the specificity of m⁶A enrichment in the UTRs, consistent with the known preference of m⁶A for UTRs in mammalian mRNAs (Supplementary Fig. S1D). MACS2 peak calling identified ~5918 high-confidence m^6^A-modified transcripts across three biological replicates (Fig. 1A). This demonstrates the widespread presence of m^6^A modifications within the mouse NK cell transcriptome.

Consistent with established patterns observed in other cell types, m^6^A peaks were significantly enriched with the consensus motif “GGACU”across all replicates (Fig. 1B). Metagene analysis further delineated the positional enrichment of m^6^A peaks. These peaks were predominantly localized within coding sequences (CDS) and 3’ untranslated regions (3’ UTRs), with the highest density clustered proximally to stop codons (Fig. 1C).

To further investigate m^6^A functions in NK cells, we performed RNA-sequencing (RNA-seq) on three distinct wild-type (WT) NK cell developmental populations: NKP, iNK, and mature NK cells (mNK). Integrating these transcriptomic profiles with our global m^6^A map revealed that developmental transitions, particularly during NKP-to-iNK stage, coincided with widespread gene expression changes for m^6^A-modified transcripts. Comparative analysis indicated significantly more pronounced transcriptomic alterations (both upregulation and downregulation) occurred between the NKP and iNK stages than between the iNK and mNK stages (Fig. 1D). Pearson correlation analysis confirmed a closer transcriptional relationship between iNK and mNK cells than between either population and NKP cells (Fig. 1E), reinforcing the significance of the NKP-to-iNK transition.

Focusing on this key developmental transition (NKP vs. iNK), differential expression analysis (DESeq2/DEG analysis) identified 2443 upregulated and 2098 downregulated transcripts. Among these developmentally regulated genes, a substantial subset (1136 upregulated; 849 downregulated) were also m^6^A-modified (Fig. 1F), suggesting potential post-transcriptional regulation by m^6^A during early NK cell commitment and maturation. In contrast, the transcriptional shift between iNK and mNK stages was less extensive, yielding 490 upregulated and 814 downregulated transcripts. Of these, 282 upregulated and 337 downregulated genes exhibited m^6^A modification (Fig. 1G).

To delineate functional pathways governed by m^6^A-modified mRNAs during NK cell development, we conducted Kyoto Encyclopedia of Genes and Genomes (KEGG) pathway enrichment analysis on differentially expressed m^6^A-marked transcripts across key developmental transitions. Focusing on the critical NKP-to-iNK maturation stage, upregulated m^6^A-modified transcripts were significantly enriched in pathways essential for effector function acquisition, including Natural killer cell-mediated cytotoxicity, Apoptosis, Cytokine-cytokine receptor interactions, and JAK-STAT signaling. Conversely, downregulated m^6^A-modified transcripts in iNK versus NKP cells exhibited enrichment in pathways associated with early lineage commitment and progenitor activity, including Hematopoietic cell lineage, Cytokine-cytokine receptor interactions, MAPK signaling, and NF-κB signaling (Fig. 1H).

Despite minimal transcriptional differences between mNK and iNK cells, upregulated m^6^A-modified transcripts in mNK cells exhibited significant enrichment in pathways critical for effector function refinement, including lysosomal function, apoptosis, and NK cell-mediated cytotoxicity. Conversely, downregulated m^6^A-modified transcripts were linked to pathways modulating developmental signaling and inflammatory priming: NF-κB signaling, Notch signaling, JAK-STAT signaling, and cytokine-cytokine receptor interactions. Collectively, these data demonstrate that m^6^A modification likely fine-tunes early-stage NKP-to-iNK differentiation and terminal NK cell competence (Fig. 1I).

### *Mettl14* genetic ablation at the NKP stage causes severe NK cell lymphopenia

**Fig. 2:**
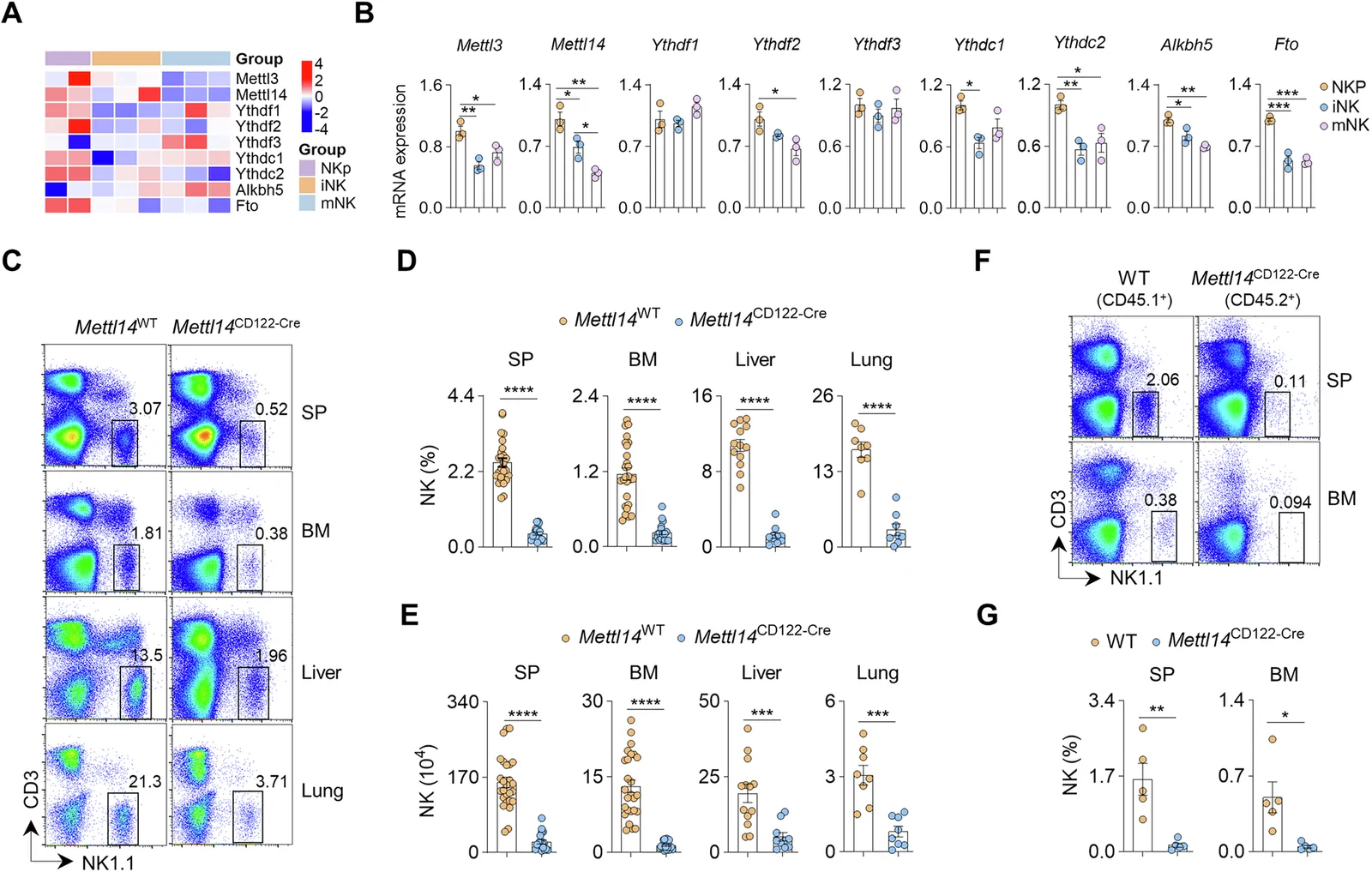
*Mettl14* genetic ablation at the NKP stage causes severe NK cell lymphopenia. **A** Heatmap of m⁶A regulator expression (writers, readers, and erasers) in WT NKP, iNK, and mNK cells. **B** RT-PCR analysis of m⁶A regulator expression in WT NK cell subsets normalized to NKP cells (*n* = 3 mice/group). Representative flow cytometric (FCM) profiles (**C**), frequencies (**D**) and absolute numbers (**E**) of NK cells in spleen (SP), bone marrow (BM), liver, and lung of *Mettl14*^WT^ and *Mettl14*^CD122-Cre^ (*n* = 8–26 mice/group). FCM analysis (**F**) and quantification (**G**) of donor-derived WT (CD45.1⁺) and *Mettl14*^CD122-Cre^ (CD45.2⁺) NK cells in the SP and BM of competitive chimera mice 8 weeks after BM reconstitution (*n* = 5 mice/group). Data are representative of three independent experiments (**B**, **F** and **G**) or pooled from at least three independent experiments (**D** and **E**). Data are presented as means ± SEM. Statistical significance was determined by one-way ANOVA (**B**) or two-tailed unpaired Student’s *t* tests (**D**, **E**, **G**). *P < 0.05; **P < 0.01; ***P < 0.001; ****P < 0.0001.

Integrated analysis of MeRIP-seq and RNA-seq data across three NK cell developmental populations (NKP, iNK, mNK) revealed pronounced expression changes in m^6^A-modified transcripts specifically during the NKP-to-iNK transition (Fig. 1D). This finding suggests that m^6^A RNA modification critically regulates early NK cell development. To validate this hypothesis, we quantified the expression of m^6^A regulatory machinery components (writers, readers, erasers) in murine NK cell subsets using transcriptomic data. Notably, m^6^A machinery genes, including writers (*Mettl3*, *Mettl14*), readers (*Ythdf1*, *Ythdf2*, *Ythdc1*, *Ythdc2*), and the eraser *Fto*, exhibited peak expression in NKP cells compared to iNK and mNK populations (Fig. 2A). This stage-specific enrichment was further confirmed through independent validation using quantitative real-time PCR (qRT-PCR) (Fig. 2B).

The METTL3-METTL14 complex constitutes the core catalytic module for RNA m^6^A methylation. Prior studies established that ablation of METTL14, a scaffold component essential for methyltransferase complex integrity, completely abolishes global m^6^A modification [17]. To dissect the role of m^6^A in early NK cell development, we generated conditional knockout mice (*Mettl14*^fl/fl^CD122-Cre^+^, hereafter *Mettl14*^CD122-Cre^) by crossing *Mettl14*-floxed mice (*Mettl14*^fl/fl^) with CD122-Cre knock-in mice. CD122-Cre drives NK lineage-specific deletion from the NK progenitor (NKP) stage onward [33], achieving dramatic reduction of *Mettl14* mRNA in sorted NK cells (Supplementary Fig. S2A).

Compared to *Mettl14*^WT^ littermate controls (*Mettl14*^fl/fl^CD122-Cre^−^), conditional *Mettl14* deletion caused severe NK cell lymphopenia, with 80–85% reductions in NK cell frequency and absolute numbers across multiple organs, including spleen, bone marrow (BM), liver, and lung (Fig. 2C–E). To determine whether this defect stemmed from cell-intrinsic mechanisms, we performed competitive bone marrow reconstitution assays. Lethally irradiated *Rag1*^−/−^*γc*^−/Y^ immunodeficient mice (lacking mature T, B, and NK cells) were reconstituted with mixed BM from WT (CD45.1^+^) and *Mettl14*^CD122-Cre^ (CD45.2^+^) donors. Despite equivalent reconstitution efficiency of *Mettl14*-deficient hematopoietic stem cells (HSCs) in spleen and BM, NK cells derived from *Mettl14*^CD122-Cre^ BM were markedly reduced (Fig. 2F, G). This selective NK cell deficiency conclusively demonstrates that METTL14 regulates NK cell homeostasis through cell-intrinsic mechanisms.

We quantified other major immune cell populations in the spleen, bone marrow, and liver of *Mettl14*^WT^ and *Mettl14*^CD122-Cre^ mice. CD122-Cre-mediated *Mettl14* deletion minimally affected conventional lymphocyte development, and caused only slight reductions in the frequencies and absolute numbers of T and B cells across all tissues (Supplementary Fig. S2B, C). However, IL-15 dependent innate lymphoid populations were significantly reduced in *Mettl14*-knockout mice. NKT cell frequencies and absolute numbers were markedly decreased in the thymus and spleen, with only a reduction in frequency observed in the liver (Supplementary Fig. S2D, E). Similarly, hepatic conventional NK (cNK) cells showed decreases in both frequency and absolute number, whereas hepatic ILC1s exhibited a significant reduction only in absolute counts (Supplementary Fig. S2F–H).

### METTL14 deficiency disrupts the transition of NKP/iNK to mNK

**Fig. 3:**
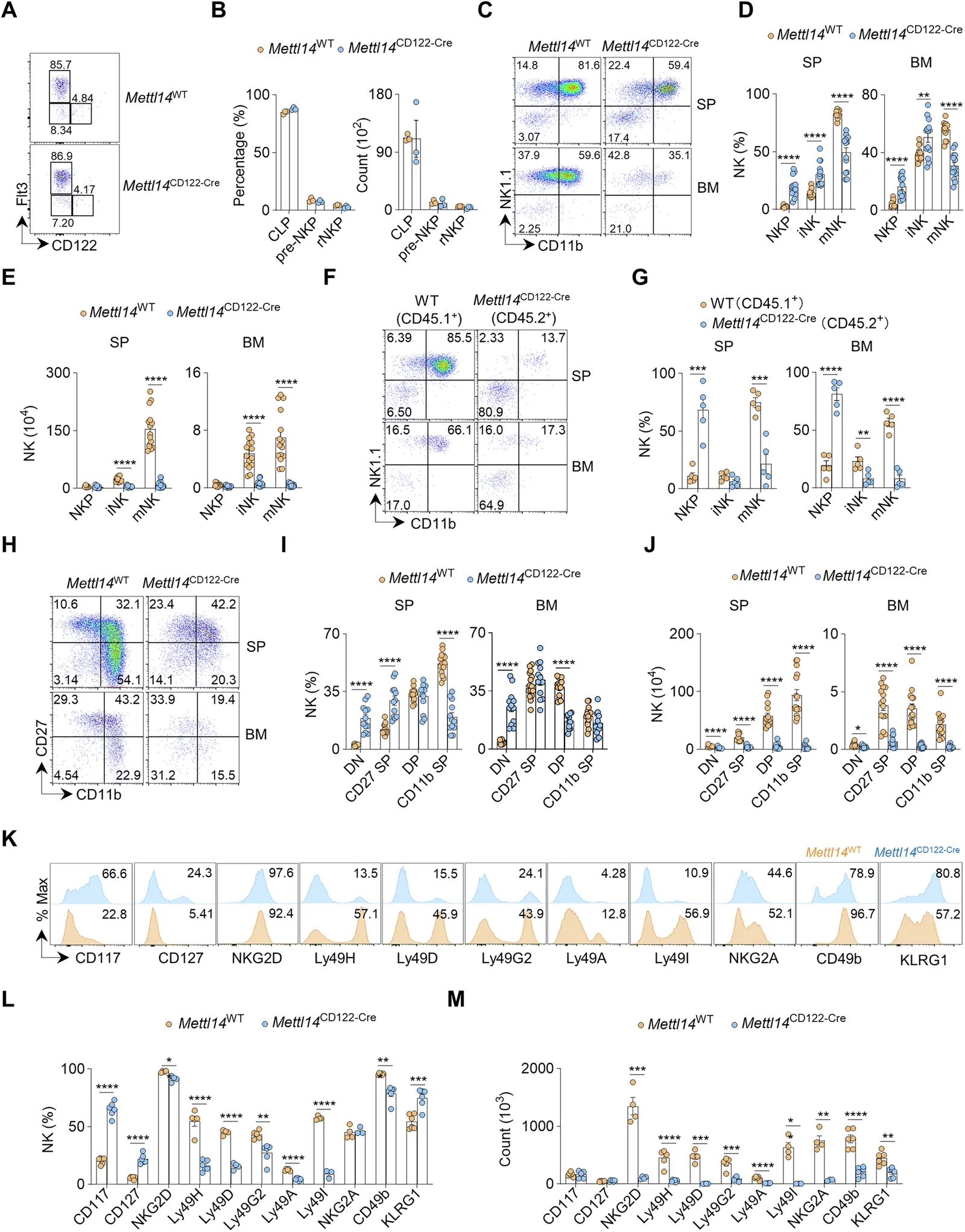
METTL14 deficiency disrupts the transition of NKP/iNK to mNK. Representative FCM profiles (**A**), frequencies, and absolute numbers (**B**) of CLP (Lin⁻CD27^+^CD244^+^ CD127^+^Flt3^+^CD122⁻), Pre-NKP (Lin⁻CD27^+^CD244^+^CD127^+^Flt3^+^CD122⁻), and rNKP (Lin⁻CD27^+^CD244^+^ CD127^+^ Flt3⁻CD122^+^) cells in BM of *Mettl14*^WT^ and *Mettl14*^CD122-Cre^ mice (*n* = 3 mice/group). Representative FCM profiles (**C**), frequencies (**D**), and absolute numbers (**E**) of NKP, iNK and mNK cells in SP and BM of *Mettl14*^WT^ and *Mettl14*^CD122-Cre^ mice (*n* = 14–15 mice/group). FCM analysis (**F**) and quantification (**G**) of donor-derived WT (CD45.1⁺) and *Mettl14*^CD122-Cre^ (CD45.2⁺) NK cells in the SP and BM of competitive chimera mice 8 weeks after BM reconstitution (*n* = 5 mice/group). Representative FCM profiles (**H**), frequencies (**I**), and absolute numbers (**J**) of four-stage development, including CD27⁻CD11b⁻ (DN), CD27^+^CD11b⁻ (CD27 SP), CD27^+^CD11b^+^ (DP), and CD27⁻CD11b^+^ (CD11b SP) of the gated CD3⁻NK1.1^+^ NK cells in the spleen and bone marrow of *Mettl14*^WT^and *Mettl14*^CD122-Cre^ mice (*n* = 12–15 mice/group). FCM analysis (**K**), frequencies (**L**), and absolute numbers (**M**) of development-related receptor positive NK cells in the spleen of the indicated mice (*n* = 4-6 mice/group). Data are representative of three independent experiments (**B**, **G**, **L**, and **M**) or pooled from at least three independent experiments (**D**, **E**, **I**, and **J**). Data are presented as means ± SEM. Statistical significance was determined by two-tailed unpaired Student’s *t* tests. *P < 0.05; **P < 0.01; ***P < 0.001; ****P < 0.0001.

NK cell development proceeds from common lymphoid progenitors (CLPs) through pre-NKPs, refined NKPs (rNKPs), NKPs, iNK cells, to mNK cells [4]. To define the impact of METTL14 ablation on this pathway, we analyzed developmental stages in *Mettl14*^CD122-Cre^ mice. Evaluation of bone marrow progenitors revealed no significant alterations in CLP, pre-NKP, or rNKP populations compared to wild-type controls (Fig. 3A, B). However, analysis of later developmental stages uncovered a profound defect. In spleen and bone marrow, *Mettl14*^CD122-Cre^ mice exhibited significantly increased frequencies of NKPs and iNK cells, but decreased frequencies of mNK cells (Fig. 3C, D). Despite the elevated frequency of NKPs, their absolute numbers remained normal. Conversely, absolute numbers of iNK and mNK cells were substantially reduced in both tissues (Fig. 3E). This differentiation defect was cell-intrinsic, as evidenced by bone marrow chimera experiments: The CD45.2⁺ compartment derived from *Mettl14*^CD122-Cre^ mice recapitulated the phenotype of increased NKP frequency and decreased mNK frequency in both the spleen and bone marrow. However, the reduction in iNK frequency was observed only in the bone marrow when compared with CD45.1⁺ WT-derived cells (Fig. 3F, G). Collectively, the pronounced accumulation of iNK cells (by frequency) alongside a severe reduction in mature iNK and mNK cells (by number) demonstrates that METTL14 ablation blocks NK cell differentiation at the transition from NKP/iNK to mNK cells, shortly after the acquisition of CD122 expression.

We next assessed NK cell maturation using the canonical CD27 and CD11b-defined stages: DN (CD27^−^CD11b^−^), CD27 SP (CD27^+^CD11b^−^), DP (CD27^+^CD11b^+^), and CD11b SP (CD27^−^CD11b^+^) [34]. *Mettl14* deficiency severely disrupted this maturation program. In the spleen of *Mettl14*^CD122-Cre^ mice, we observed a significant accumulation of immature DN and CD27 SP subsets and a concomitant reduction in the mature CD11b SP population in the spleen (Fig. 3H, I). A parallel skewing toward immature subsets was confirmed in the CD45.2^+^ compartment of competitive bone marrow chimeras (Supplementary Fig. S2I), establishing the cell-intrinsic nature of this defect. In the bone marrow, *Mettl14*-deficient NK cells similarly exhibited an increase in the immature DN subset and a decrease in the DP population (Fig. 3H, I). Critically, despite the relative accumulation of certain immature subsets by frequency, the absolute numbers of NK cells at all maturation stages were markedly reduced in both spleen and bone marrow of knockout mice (Fig. 3J). This global reduction underscores a profound deficit in NK cell development.

Further surface phenotyping revealed the molecular underpinnings of this maturation arrest. Splenic *Mettl14*-deficient NK cells displayed retained expression of early developmental markers, with increased proportions of CD117^+^ and CD127^+^ cells (Fig. 3K–M). Conversely, the acquisition of late-stage maturation markers was severely impaired, as evidenced by a diminished proportion of CD49b^+^ NK cells and a stark reduction in the expression of individual Ly49 receptors (Fig. 3K–M). Collectively, these data demonstrate that METTL14 is essential for coordinating the transition from immature to functionally mature NK cells, with its loss enforcing a blockade at the late stages of maturation.

### METTL14 coordinates the transcriptional program for NKP to iNK transition

Although *Mettl14*-deficient mice maintained normal numbers of NKPs (Fig. 3E), the severe reduction in iNK and mNK populations pointed to a potential developmental blockade at the NKP-to-iNK transition. To investigate the underlying molecular mechanism, we performed RNA-seq on sorted NKPs from *Mettl14*^CD122-Cre^ and wild-type mice.

**Fig. 4:**
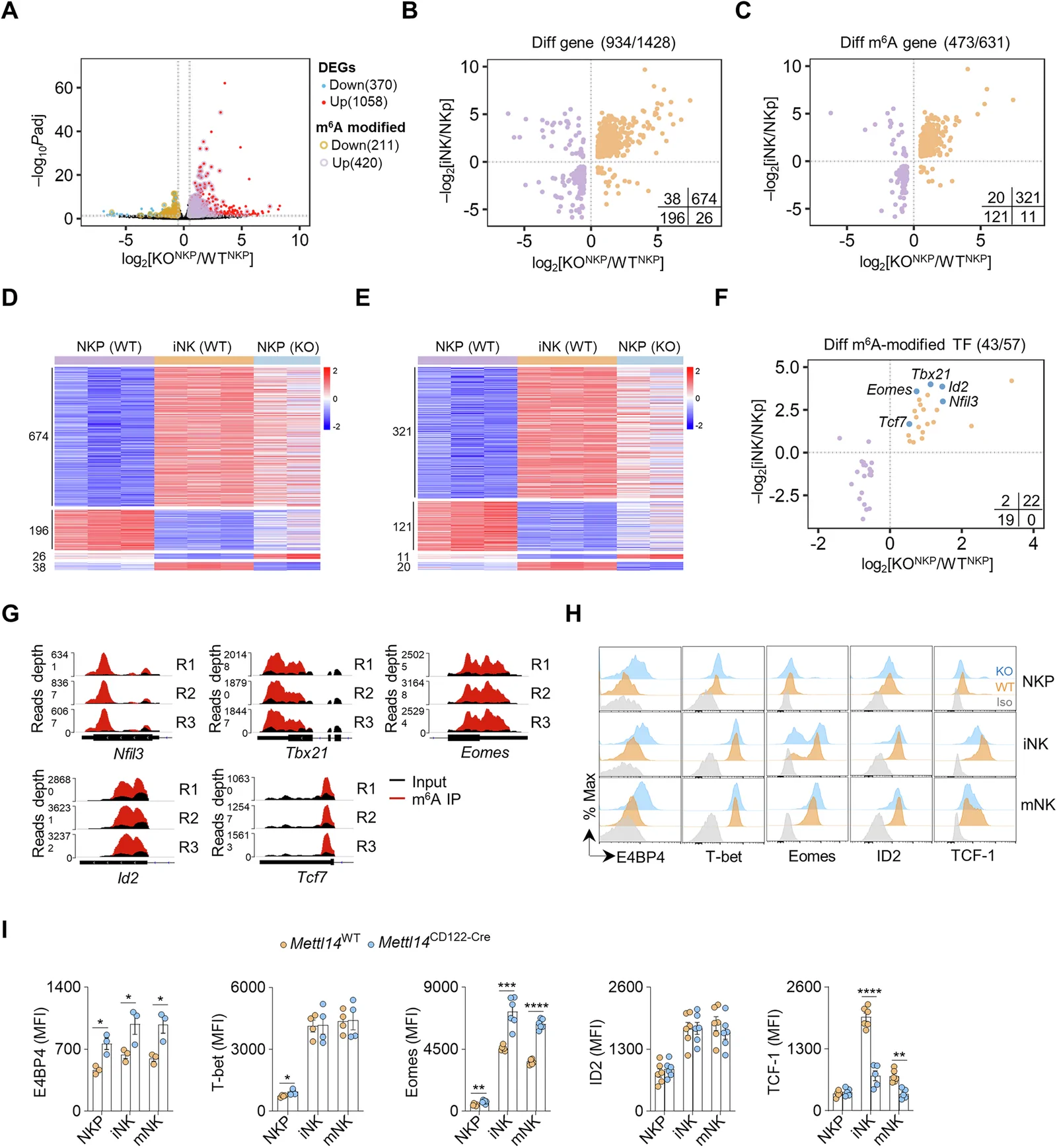
METTL14 coordinates the transcriptional program for NKP to iNK transition. **A** Volcano plot of differentially expressed transcripts in *Mettl14*^CD122-Cre^ versus *Mettl14*^WT^ NKP cells (adjusted *P* ≤ 0.05; log_2_ fold-change ≥0.5). m^6^A-modified genes are marked with circles. Volcano plots of all DEGs (**B**), m⁶A-modified DEGs (**C**) and differentially expressed m⁶A-modified transcription factors (**F**) in *Mettl14*^CD122-Cre^ versus *Mettl14*^WT^ NKP cells (adjusted *P* ≤ 0.05; log_2_ fold-change ≥0.5). Heatmaps of all DEGs (**D**) and m⁶A-modified DEGs (**E**) expression across *Mettl14*^WT^ NKP, *Mettl14*^WT^ iNK, and *Mettl14*^CD122-Cre^ NKP cells (adjusted *P* ≤ 0.05; log_2_ fold-change ≥0.5). **G** m⁶A peak enrichment in 3’UTRs of key regulators (*Nfil3*, *Tbx21*, *Eomes*, *Id2*, *Tcf7*) from WT NK cell meRIP-seq. R1-R3 represent biological replicates. **H**, **I** Representative FCM profiles of E4BP4, T-bet, Eomes, ID2, TCF-1 (**H**) and corresponding mean fluorescence intensity (MFI) in splenic NK cell subsets from *Mettl14*^WT^ and *Mettl14*^CD122-Cre^ mice (*n* = 3–6 mice/group). Data are representative of at least three independent experiments (**H**, **I**). Data are presented as means ± SEM. Statistical significance was determined by two-tailed unpaired Student’s *t* tests. *P < 0.05; **P < 0.01; ***P < 0.001; ****P < 0.0001.

We identified 1428 differentially expressed genes (DEGs) in *Mettl14*-deficient NKPs, with a strong overrepresentation of m^6^A-modified transcripts (420 upregulated and 211 downregulated; Fig. 4A). Strikingly, ~65% (934/1428) of these DEGs are normally dynamically regulated during the NKP-to-iNK transition in wild-type cells (Fig. 4B). This included nearly 75% (473/631) of all differentially expressed, m^6^A-modified transcripts (Fig. 4C).

The transcriptional landscape of *Mettl14*-deficient NKPs exhibited a specific pattern of precocious activation and silencing. Most genes (674/934) that are normally upregulated during the NKP-to-iNK transition, including 321 m^6^A-modified genes, were highly expressed in *Mettl14*-deficient NKPs. Conversely, most genes (196/934) that are normally downregulated during this transition, including 121 m^6^A-modified transcripts, showed low expression in the knockout NKPs (Fig. 4D, E).

This coordinated dysregulation extended to 57 transcription factors (TFs) critical for NK cell differentiation, 43 of which were m^6^A-modified (Fig. 4F). Notably, key differentiation regulators, including *Nfil3* (E4BP4), *Tbx21* (T-bet), *Eomes*, *Id2*, and *Tcf7* (TCF-1), were all m^6^A-modified and exhibited significantly altered expression in *Mettl14*-deficient NKPs (Fig. 4F, G). Flow cytometry confirmed elevated protein levels of E4BP4, T-bet, and Eomes in *Mettl14*-deficient NKPs, with E4BP4 and Eomes also increased in iNK and mNK cells, whereas TCF-1 was downregulated in later stages (Fig. 4H, I).

These results demonstrate that METTL14-mediated m^6^A methylation is essential for orchestrating the precise transcriptional timing required for the NKP to iNK transition, and its loss disrupts the coordinated activation and repression of a core differentiation program.

### METTL14 deficiency drives hyperproliferation associated with metabolic stress and DNA damage response

**Fig. 5:**
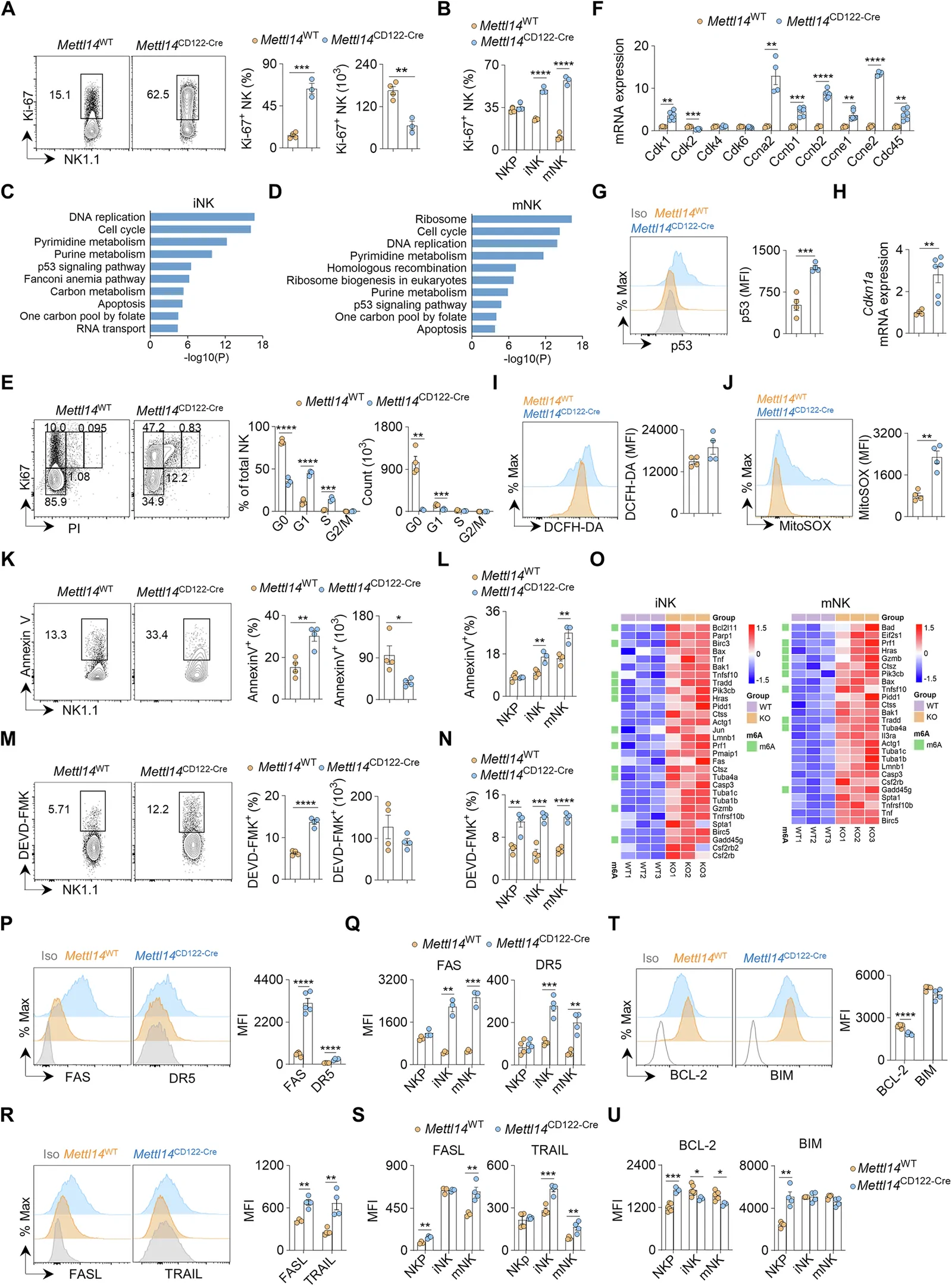
METTL14 deficiency induces hyperproliferation and enhances apoptosis in NK cells. **A** Representative FCM plots (left), frequencies, and absolute numbers (right) of Ki67⁺ cells in splenic NK cells (*n* = 3–4 mice/group). **B** Ki67⁺ frequencies in NK cell subsets (*n* = 3–4 mice/group). KEGG pathway enrichment of upregulated DEGs in iNK (**C**) and mNK (**D**) cells from *Mettl14*^CD122-Cre^ versus *Mettl14*^WT^ mice (adjusted *P* ≤ 0.05; log₂ fold-change ≥0.5). **E** Representative FCM profiles (left), frequencies, and absolute numbers (right) of cell cycle phases, including G0 (Ki67^−^PI^−^), G1 (Ki67^+^PI^−^), S (Ki67^+^PI^low^), and G2/M (Ki67^+^PI^hi^) in splenic NK cells (*n* = 3–4 mice/group). **F** RT-PCR analysis of indicated cell cycle regulators in splenic NK cells (*n* = 4–6 mice/group). **G** Representative FCM profiles of p53 (left) and corresponding MFI (right) in splenic NK cells (*n* = 4 mice/group). **H** RT-PCR analysis of *Cdkn1a* expression in splenic NK cells (*n* = 4–6 mice/group). Cytosolic ROS (DCFH-DA) (**I**) and Mitochondrial ROS (MitoSOX) (**J**) in splenic NK cells (*n* = 4 mice/group). Representative FCM plots (left), frequencies, and absolute numbers (right) of Annexin V^+^ (**K**) and DEVD-FMK^+^ (**M**) cells in splenic NK cells (*n* = 4 mice/group). Annexin V^+^ (**L**) and DEVD-FMK^+^ (**N**) frequencies in NK cell subsets (*n* = 3–4 mice/group). **O** Heatmaps depicting upregulated genes related to apoptosis signaling pathways in *Mettl14*-deficient iNK (left) or mNK (right) cells compared to relative WT controls (adjusted *P* ≤ 0.05; log_2_ fold-change). Representative FCM plots (left) and MFI (right) of FAS and DR5 (**P**), FASL and TRAIL (**R**), BCL-2 and BIM (**T**) in splenic NK cells from the indicated mice (*n* = 3–6 mice/group). MFI of FAS and DR5 (**Q**), FASL and TRAIL (**S**), BCL-2 and BIM (**U**) in NK cell subsets (*n* = 3–6 mice/group). Data are representative of at least three independent experiments. Data are presented as means ± SEM. Statistical significance was determined by two-tailed unpaired Student’s *t* tests. *P < 0.05; **P < 0.01; ***P < 0.001; ****P < 0.0001.

Given the pronounced NK cell lymphopenia in *Mettl14*^CD122-Cre^ mice, we initially assessed cell proliferation. Contrary to expectations, Ki-67 staining revealed a two- to five-fold increase in the frequency of Ki-67⁺ cells within both iNK and mNK subsets at steady state (Fig. 5A, B). To resolve this paradox, we performed RNA-seq on sorted naïve NK cells. Gene set enrichment analysis (GSEA) and KEGG pathway analysis of the transcriptomic data revealed significant enrichment of signatures related to cell cycle, DNA replication, and nucleotide metabolism in *Mettl14*-deficient iNK and mNK cells (Supplementary Fig. S3A–E; Fig. 5C, D).

Consistent with the transcriptomic data, cell-cycle analysis revealed a higher percentage of *Mettl14*-deficient NK cells in G1 and S phases, and a corresponding decrease in G0 (Fig. 5E), indicating accelerated cell cycle entry and progression. This was further supported by the substantial upregulation of key cell cycle promoters, e.g., *Cdk1*, *Ccna2*, *Ccnb1*, *Ccnb2*, *Ccne1*, *Ccne2* and *Cdc45*, at the mRNA level, although *Cdk2* expression was notably reduced (Fig. 5F).

This hyperproliferative state was coupled with clear signs of cellular stress. We observed significant enrichment of the p53 signaling pathway (Fig. 5C, D; Supplementary Fig. S3C, D), accompanied by increased p53 protein and its transcriptional target *Cdkn1a* (p21) mRNA (Fig. 5G, H), suggesting activation of a DNA damage response. GSEA also indicated enhanced activity in DNA repair pathways, including homologous recombination, base excision repair and mismatch repair (Supplementary Fig. S3C, D). In line with this, our RNA-seq data revealed significant upregulation of genes encoding key machinery involved in base excision repair (*Pcna*, *Pole*, *Lig1*, *Fen1*, *Tdg*, *Neil3*, *Apex1*, *Ung*) and mismatch repair (*Msh6*, *Exo1*, *Rfc5*, *Rfc3*) in *Mettl14*-deficient NK cells (data not shown). As a potential source of this genomic stress, we measured reactive oxygen species (ROS) and found that *Mettl14*-deficient NK cells exhibited significant accumulation of mitochondrial superoxide (Fig. 5J), despite normal levels of cytosolic ROS (Fig. 5I). The expression of genes encoding the antioxidant enzymes GPX4 and GPX8 was significantly downregulated in the absence of *Mettl14* (data not shown), which may render the cells more susceptible to ROS-induced cell death.

Collectively, these data demonstrate that METTL14 deficiency induces a hyperproliferative phenotype in NK cells, which is paradoxically linked to metabolic oxidative stress and the activation of p53-mediated checkpoint signaling, likely contributing to the overall homeostatic defect.

### METTL14 deficiency triggers apoptosis through convergent extrinsic and intrinsic pathways

Given the observed mitochondrial ROS accumulation and p53 pathway activation, we hypothesized that *Mettl14*-deficient NK cells undergo enhanced apoptosis. Indeed, freshly isolated *Mettl14*^CD122-Cre^ NK cells exhibited an approximately two-fold increase in Annexin V⁺ cells and significantly elevated caspase activity (Fig. 5K, M). This pro-apoptotic phenotype was most pronounced within the iNK and mNK subsets (Fig. 5L, N), corroborated by significant enrichment of apoptosis-related KEGG pathways in these populations (Fig. 5C, D).

Transcriptomic analysis revealed broad upregulation of apoptotic genes in *Mettl14*-deficient iNK and mNK cells (Fig. 5O). This included classical p53 transcriptional targets, such as the BH3-only proteins *Pmaip1* (Noxa), *Bax*, and *Bak1*, death receptors *Fas* and *Tnfrsf10b* (DR5), and the adapter *Pidd1*. Furthermore, death ligands *Tnf* (TNF-α) and *Tnfsf10* (TRAIL) were upregulated alongside their receptors. Additional pro-apoptotic genes not directly regulated by p53, including *Bcl2l11* (Bim), *Tradd*, and *Casp3*, were also elevated, as were transcripts for the lysosomal enzymes *Ctss* and *Ctsz* (Fig. 5O), which can amplify caspase activation via mitochondrial damage.

This transcriptional profile was validated at the protein level. Flow cytometry confirmed increased surface expression of FAS, DR5, and TRAIL on total, iNK, and mNK cells from *Mettl14*^CD122-Cre^ mice, alongside elevated FASL, particularly on NKP and mNK subsets (Fig. 5P–S). Concurrently, while BIM protein levels were unchanged, the anti-apoptotic protein BCL-2 was significantly downregulated (Fig. 5T, U).

Collectively, these results demonstrate that METTL14 deficiency induces concurrent activation of both the extrinsic (death receptor/ligand) and intrinsic (mitochondrial/BCL-2 family) apoptotic pathways, culminating in the preferential loss of iNK and mNK cells probably through FAS-FASL, TRAIL-DR5, and TNF-α-TNFR1 mediated mechanisms.

### METTL14 deficiency induces a hypermetabolic state in NK cells via mTORC1 activation

**Fig. 6:**
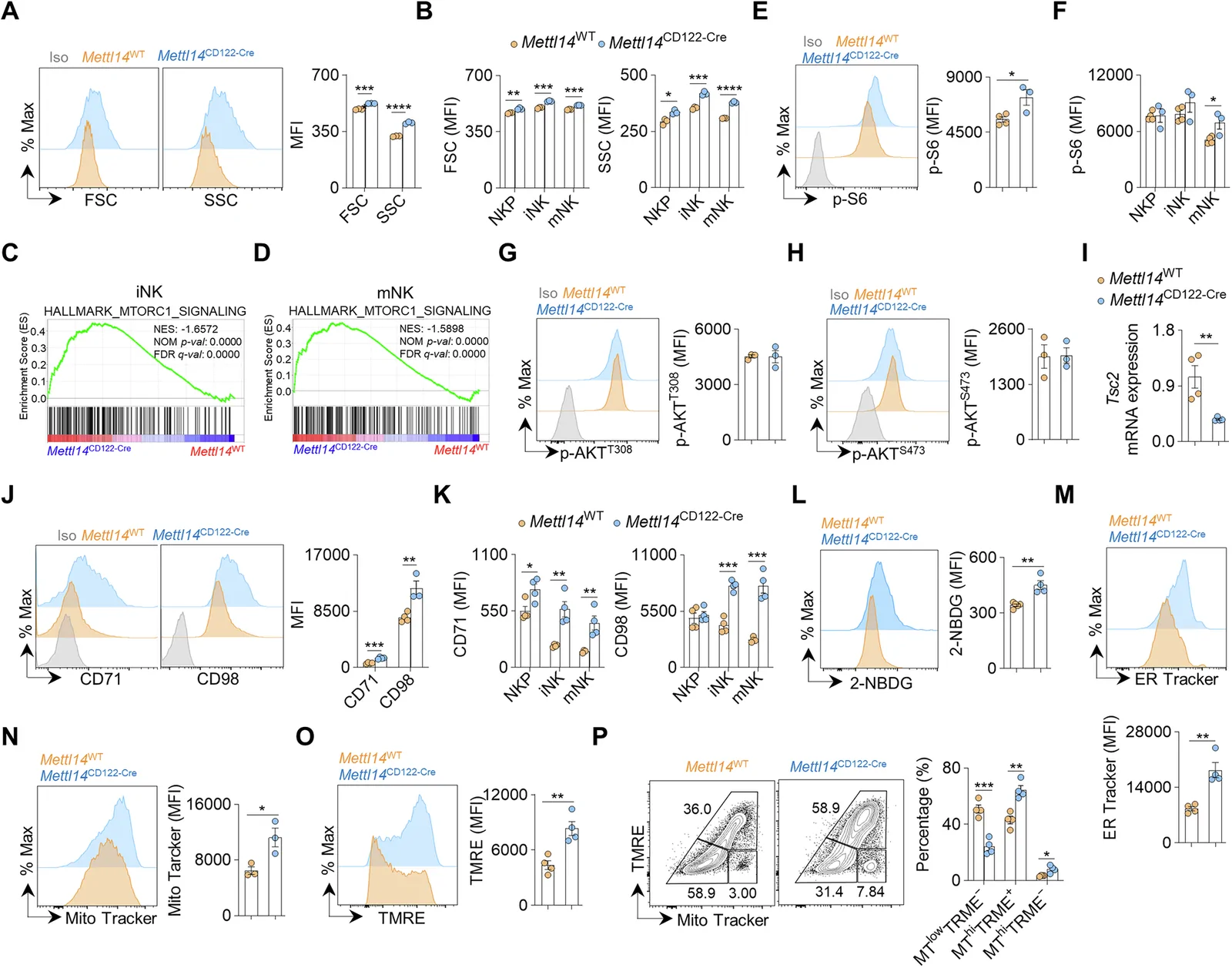
METTL14 deficiency induces a hypermetabolic state in NK cells via mTORC1 activation. **A** Representative FCM profiles (left) and mean fluorescence intensity (MFI, right) depicting the cell size (FSC) and granularity (SSC) of freshly isolated splenic NK cells (*n* = 3 mice/group). **B** MFI of FSC (left) and SSC (right) of NK cell subsets (*n* = 3 mice/group). Gene Set Enrichment Analysis (GSEA) of “HALLMARK_MTORC1_SIGNALING” of iNK (**C**) and mNK (**D**) cells from *Mettl14*^CD122-Cre^ versus *Mettl14*^WT^ mice. mTORC1, mammalian target of rapamycin complex 1. Representative FCM plots (left) of p-S6 (**E**), p-AKT^T308^ (**G**), p-AKT^S473^ (**H**), CD98, and CD71 (**J**) and corresponding MFI (right) in splenic NK cells (*n* = 3–4 mice/group). MFI of pS6 (**F**), CD98, and CD71 (**K**) in NK cell subsets (*n* = 3–4 mice/group). **I** RT-PCR analysis of *Tsc2* expression in splenic NK cells (*n* = 4 mice/group). **L** Representative FCM profiles (left) and MFI (right) depicting 2-NBDG uptake by total splenic NK cells (*n* = 4–5 mice/group). Endoplasmic reticulum mass (ER Tracker Green) (**M**), Mitochondrial mass (MitoTracker Green) (**N**) and membrane potential (TMRE incorporation) (**O**) of the splenic NK cells (*n* = 3–4 mice/group). **P** Representative FCM plots (left) and frequencies (right) of TMRM versus MitoTracker Green of the splenic NK cells from the indicated mice (*n* = 4 mice/group). Data are representative of at least three independent experiments. Data are presented as means ± SEM. Statistical significance was determined by two-tailed unpaired Student’s *t* tests. *P < 0.05; **P < 0.01; ***P < 0.001; ****P < 0.0001.

To support their heightened proliferation and effector potential, NK cells undergo extensive metabolic remodeling. Consistent with this, GSEA revealed significant enrichment of anabolic pathways, including nucleotide, amino acid, glycerolipid, and glycolytic biosynthesis, in *Mettl14*-deficient NK cells (Supplementary Fig. S3C, D). This transcriptional signature was corroborated by increased cell size and granularity, established proxies of metabolic activation, which were elevated across all maturation stages in knockout cells (Fig. 6A, B).

Mitochondria are essential hubs that orchestrate cellular metabolism and have recently been shown to be regulated by mTOR signaling [35, 36]. We next investigated whether the metabolic shift was linked to mTOR signaling, a central regulator of cellular metabolism. GSEA indicated strong positive enrichment of mTORC1-related gene sets in both *Mettl14*-deficient iNK and mNK cells (Fig. 6C, D). Accordingly, phosphorylation of the mTORC1 substrate S6 (p-S6) was significantly elevated, particularly in mNK subsets (Fig. 6E, F). While AKT phosphorylation at Ser473 and Thr308 remained unchanged (Fig. 6G, H), we observed a marked downregulation of *Tsc2*, a key negative regulator of mTORC1 (Fig. 6I), suggesting a mechanism for mTORC1 hyperactivation independent of canonical AKT signaling.

This metabolic rewiring was functionally evident in several readouts: Surface expression of the transferrin receptor CD71 and the amino acid transporter CD98 was significantly upregulated in *Mettl14*-deficient splenic NK cells (Fig. 6J, K). Glucose uptake, measured by 2-NBDG incorporation, was enhanced (Fig. 6L). Endoplasmic reticulum abundance, visualized by ER-Tracker Green, was increased (Fig. 6M), indicating elevated biosynthetic capacity. Mitochondrial analysis further revealed that *Mettl14*-deficient NK cells possessed higher mitochondrial mass (MitoTracker Green) and membrane potential (TMRM) than wild-type cells (Fig. 6N, O). A substantial fraction of knockout cells maintained both high mitochondrial content and elevated membrane potential at steady state (Fig. 6P), indicative of enhanced mitochondrial fitness.

In summary, METTL14 deficiency drives NK cells into a hypermetabolic state characterized by mTORC1 activation, increased nutrient uptake, and augmented mitochondrial function. While supporting effector potential, this chronically activated metabolic profile ultimately disrupts NK cell development and survival in vivo, underscoring the role of METTL14 in calibrating metabolic fitness to ensure homeostatic balance.

### METTL14 deficiency induces a hyperactive phenotype and enhances antitumor immunity in NK cells

**Fig. 7:**
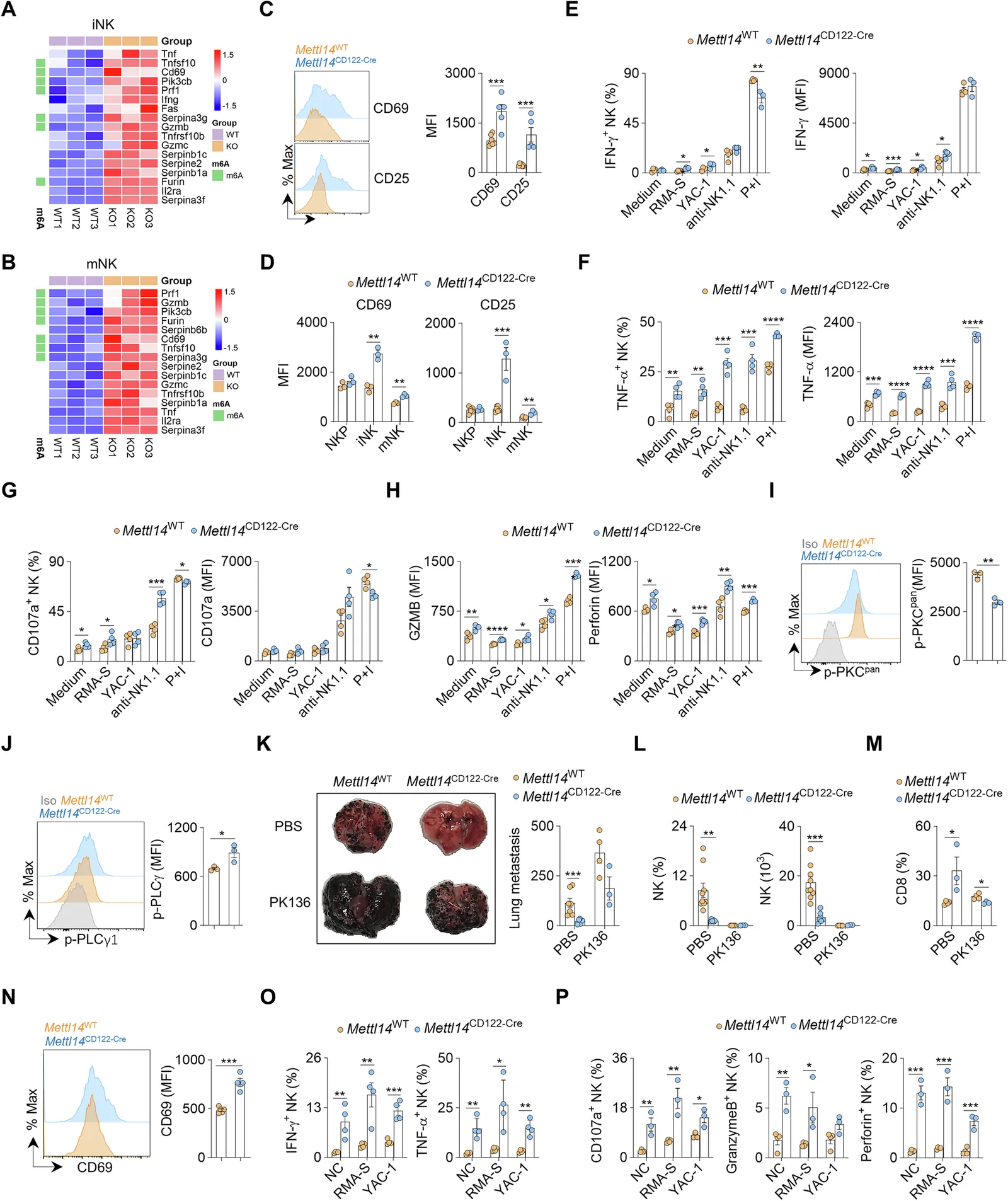
METTL14 deficiency induces a hyperactive phenotype and enhances antitumor immunity in NK cells. Heatmap of a subset of upregulated signature genes associated with NK cell activation in splenic iNK (**A**) and mNK (**B**) cells from *Mettl14*^CD122-Cre^ mice compared to *Mettl14*^WT^ mice. **C** Representative FCM profiles of CD69 and CD25 (left) and corresponding MFI (right) on freshly isolated splenic NK cells (*n* = 5–7 mice/group). **D** MFI of CD69 and CD25 on freshly isolated splenic NK cell subsets (*n* = 5–6 mice/group). **E**–**H** Naive splenocytes from *Mettl14*^WT^ and *Mettl14*^CD122-Cre^ mice were either unstimulated (NC) or stimulated with RMA-S, YAC-1, plate-coated antibodies, or PMA plus ionomycin. The percentages (left) and (right) MFI of IFN-γ^+^ (**E**), TNF-α^+^ (**F**) and CD107^+^ (**G**) cells splenic NK cells were analyzed. Granzyme B (GZMB) and perforin expression (**H**) were quantified on a per-cell basis as MFI (*n* = 3–4 mice/group). Representative FCM profiles (left) of p-PKC^pan^ (**I**), p-PLCγ (**J**) and corresponding MFI (right) on freshly isolated splenic NK cells (*n* = 3 mice/group). **K** In the B16 metastasis model, the indicated mice were pretreated with or without anti-NK1.1 antibody (PK136) and then intravenously injected with B16-F10 cells (2 × 10⁵). Mice were euthanized 14 days post-inoculation. Representative lung morphology (left) and quantification (right) of total metastatic lung nodules are shown (*n* = 3–7 mice/group). Percentages and absolute numbers of NK cells (**L**) and CD8^+^ T cells (**M**) in the lungs of *Mettl14*^WT^ and *Mettl14*^CD122-Cre^ mice subjected to the B16 metastasis model (*n* = 3–9 mice/group). **N** Representative FCM profiles of CD69 (left) and corresponding MFI (right) on NK cells from the lungs of *Mettl14*^WT^ and *Mettl14*^CD122-Cre^ mice subjected to the B16 metastasis model (*n* = 4–5 mice/group). **O**, **P** the indicated mice were injected intravenously with B16-F10 (2 × 10^5^) cells. After 14 days, cells from the lungs of *Mettl14*^WT^ and *Mettl14*^CD122-Cre^ mice were either unstimulated or stimulated with the indicated tumor targets. Cumulative results show the percentages of IFN-γ^+^ and TNF-α^+^ cells (**O**), CD107a^+^ cells, GZMB^+^ cells, and perforin^+^ cells (**P**) among the total NK cells (*n* = 3–4 mice/group). Data are representative of at least three independent experiments. Data are presented as means ± SEM. Statistical significance was determined by two-tailed unpaired Student’s *t* tests. *P < 0.05; **P < 0.01; ***P < 0.001; ****P < 0.0001.

Based on the observed metabolic activation and elevated expression of FAS, FASL, and TRAIL—hallmarks of reactive NK cells, we hypothesized that METTL14 deficiency predisposes NK cells to a hyperactive state. Transcriptomic analysis supported this hypothesis, revealing upregulated expression of activation-associated genes (e.g., *Cd69*, *Il2ra*, *Furin*) and effector molecules (e.g., *Ifng*, *Tnf*, *Prf1*, *Gzmb*) in naïve *Mettl14*-deficient iNK and mNK cells (Fig. 7A, B). Flow cytometry confirmed significantly elevated surface expression of CD69 and CD25 across splenic NK cell subsets under steady-state conditions (Fig. 7C, D).

Functionally, METTL14-null NK cells exhibited heightened activation even without stimulation, as evidenced by increased frequencies of TNF-α⁺ and CD107a⁺ cells, along with elevated per-cell expression of IFN-γ, TNF-α, granzyme B, and perforin (Fig. 7E–H). Upon stimulation with RMA-S or YAC-1 tumor cells or plate-bound anti-NK1.1, *Mettl14*-deficient NK cells consistently showed enhanced cytokine production, degranulation, and cytotoxic molecule release (Fig. 7E–H). In contrast, PMA/ionomycin stimulation led to reduced proportions of IFN-γ⁺ and CD107a⁺ cells despite increased TNF-α, granzyme B, and perforin (Fig. 7E, G), a phenotype associated with impaired PKC phosphorylation and diminished cytotoxic capacity in this specific context (Fig. 7I, J).

To assess the physiological relevance of this hyperactivation, we employed a B16-F10 melanoma metastasis model. Although *Mettl14*-deficient mice exhibited reduced percentages and absolute numbers of tumor-infiltrating NK cells, they developed significantly fewer lung metastatic nodules than wild-type controls (Fig. 7K, L). PK136-mediated depletion of NK cells resulted in a substantial reduction in the number of infiltrating NK cells in the lung and a corresponding increase in metastasis in both genotypes, thereby confirming the protective role of NK cells (Fig. 7K, L). The enhanced tumor control in *Mettl14*-deficient mice correlated with increased CD8⁺ T cell infiltration in the lungs, which was abrogated upon NK cell depletion (Fig. 7M). Furthermore, lung-infiltrating NK cells from knockout mice maintained elevated CD69 expression (Fig. 7N) and showed enhanced IFN-γ/TNF-α production, CD107a exposure, and granzyme B/perforin release compared to wild-type NK cells (Fig. 7O, P).

Based on GSEA of our RNA-seq data, pathways associated with cytokine-cytokine receptor interactions were significantly upregulated (Supplementary Fig. S3C, D). In particular, expression of *Xcl1* was markedly increased in both iNK and mNK subsets, and that of *Ccl5* was elevated specifically in mNK cells (data not shown). Given that CCL5 and XCL1 are potent chemoattractants for cDC1s, and that recruitment of cDC1s into the tumor microenvironment (TME) is critical for initiating antitumor immunity, we hypothesized that enhanced secretion of these chemokines by *Mettl14*‑deficient NK cells would promote cDC1 recruitment. This in turn could improve antigen presentation and T cell activation, ultimately suppressing tumor growth.

These results collectively demonstrate that METTL14 deficiency drives NK cells into a hyperactive state, which, despite homeostatic defects, confers superior antitumor immunity through enhanced effector function and potential coordination with adaptive immune responses.

Notably, however, this sustained hyperactivation may also incur immunological costs. During long-term monitoring of *Mettl14*‑deficient mice for autoimmune phenotypes, we observed spontaneous autoimmune-like manifestations in 16‑week‑old animals, including splenomegaly and lymphadenopathy, accompanied by an increased splenocyte count (Supplementary Fig. S4A, B). These findings suggest that chronic hyperactivation of NK cells in the absence of METTL14 may contribute to immunopathology.

### METTL14 negatively regulates JAK/STAT5 and TNF signaling to maintain NK cell homeostasis

**Fig. 8:**
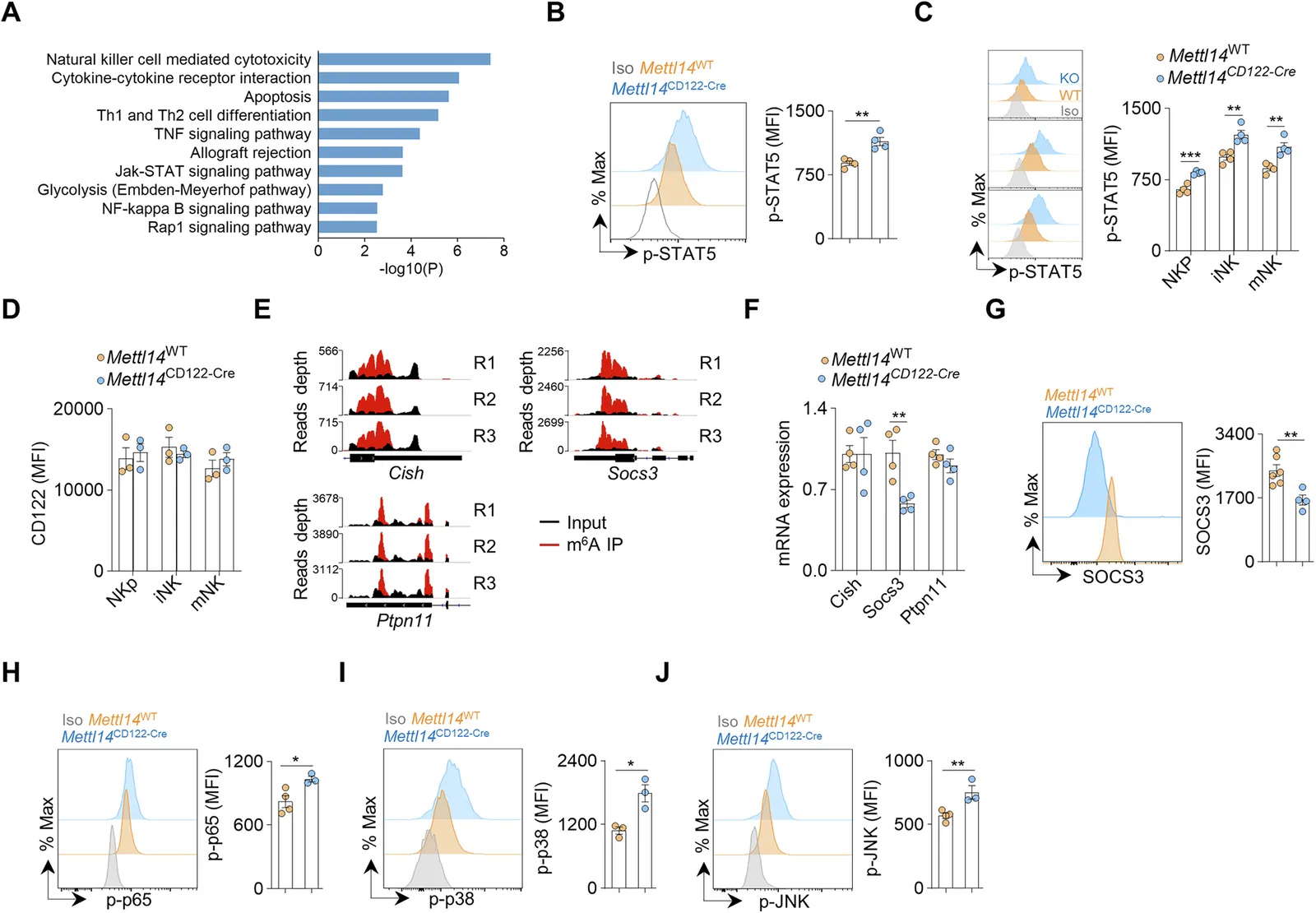
METTL14 negatively regulates JAK/STAT5 and TNF signaling to maintain NK cell homeostasis. **A** KEGG pathway enrichment of upregulated DEGs in NKP cells from *Mettl14*^CD122-Cre^ versus *Mettl14*^WT^ mice (adjusted *P* ≤ 0.05; log₂ fold-change ≥0.5). Representative FCM profiles of p-STAT5 (left) and corresponding MFI (right) on freshly isolated splenic NK cells (**B**) and NK cell subsets (**C**) (*n* = 4 mice/group). **D** MFI of CD122 on freshly isolated splenic NK cell subsets (*n* = 3 mice/group). **E** m⁶A peak enrichment in 3’UTRs of *Cish*, *Socs3*, and *Ptpn11* from WT NK cell meRIP-seq. R1-R3 represent biological replicates. **F** RT-PCR analysis of *Cish*, *Socs3*, and *Ptpn11* expression in splenic NK cells (*n* = 4 mice/group). Representative FCM profiles (left) of SOCS3 (**G**), p-p65 (**H**), p-p38 (**I**), p-JNK (**J**) and corresponding MFI (right) on freshly isolated splenic NK cells (*n* = 3–6 mice/group). Data are representative of at least three independent experiments. Data are presented as means ± SEM. Statistical significance was determined by two-tailed unpaired Student’s *t* tests. *P < 0.05; **P < 0.01; ***P < 0.001.

To elucidate the mechanisms driving spontaneous activation, metabolic dysregulation, and apoptosis in METTL14-deficient NK cells, we analyzed enriched signaling pathways. In *Mettl14*-null NKPs, upregulated genes were significantly associated with cytokine-cytokine receptor interactions, JAK-STAT signaling, and TNF signaling (Fig. 8A).

Given the central role of IL-15 in NK cell biology and the observed upregulation of IL-15–responsive genes (e.g., *Nfil3*, *Eomes*), we asked whether m^6^A methylation negatively regulates IL-15 signaling. Indeed, freshly isolated *Mettl14*-deficient NK cells exhibited elevated phosphorylation of STAT5 under steady-state conditions, despite unchanged surface expression of the IL-15 receptor subunit CD122 (Fig. 8B–D). We therefore examined key endogenous suppressors of JAK-STAT signaling and found that transcripts encoding *Cish*, *Socs3*, and *Ptpn11* all carried m^6^A modifications in NK cells (Fig. 8E). Among these, *Socs3* mRNA was significantly reduced in *Mettl14*-null NK cells, while *Cish* and *Ptpn11* levels were unaffected (Fig. 8F). Consistent with this, SOCS3 protein expression was markedly decreased in splenic NK cells from *Mettl14*^CD122-Cre^ mice (Fig. 8G). A direct link between SOCS3 depletion and heightened IL-15/JAK-STAT5 signaling was reported by Wang et al, who demonstrated that SOCS3 acts as a negative regulator of IL-15-driven NK cell maturation by promoting dephosphorylation of STAT5 [37].

TNF-α is one of the pleiotropic cytokines that play a critical role in NK cell proliferation and enhance NK cell function [38, 39]. TNFR engagement triggering p38 MAPK, JNK, and NF-κB signaling cascades implicated in cytokine production, survival, proliferation, and activation [40]. We also assessed TNF signaling, which influences NK cell proliferation, survival, and effector function. Phospho-flow analysis revealed significant increases in phosphorylated p65 (NF-κB), p38, and JNK in *Mettl14*-deficient NK cells (Fig. 8H–J), indicating hyperactivation of downstream inflammatory cascades. This dysregulation likely contributes to the observed proinflammatory phenotype, spontaneous cytokine release, and aberrant proliferation.

Together, these results establish METTL14 as a key post-transcriptional brake on IL-15 and TNF signaling, mediated in part through m^6^A-dependent suppression of SOCS3, thereby preserving NK cell quiescence and metabolic homeostasis.

### METTL14 is dispensable for late-stage NK cell maturation and function

Given the minimal transcriptomic changes and limited m^6^A modification observed during the iNK to mNK transition (Fig. 1D, E), we asked whether METTL14 is required for terminal NK cell maturation and homeostasis. To address this, we induced *Mettl14* deletion after the NKP-iNK stages using Ncr1-Cre mice (Fig. S5A). In contrast to the severe defects seen with early deletion, terminal ablation of *Mettl14* did not significantly alter the percentages or absolute numbers of NK cells in the spleen or bone marrow (Supplementary Fig. S5B–D). Moreover, *Mettl14*^Ncr1-Cre^ mice exhibited normal distribution of CD27 and CD11b-defined maturation subsets and showed no significant alterations in the NK cell receptor repertoire compared to littermate controls (Supplementary Fig. S5E–G).

Functional assessment further confirmed that late deletion of METTL14 did not impair NK cell effector responses. Upon ex vivo stimulation with RMA-S, YAC-1, or PMA/ ionomycin, *Mettl14*^Ncr1-Cre^ NK cells exhibited cytokine production and degranulation capacity comparable to wild-type controls (Supplementary Fig. S5H). In vivo functional assays revealed intact clearance of MHC class I-deficient splenocytes (Supplementary Fig. S5I) and control of melanoma metastasis (Supplementary Fig. S5J) in *Mettl14*^Ncr1-Cre^ mice.

These results demonstrate that METTL14 is dispensable for terminal NK cell maturation, homeostasis, and effector function, highlighting its stage-specific requirement during the early phases of NK cell development.

## Discussion

This study establishes the RNA methyltransferase METTL14 as a critical post-transcriptional regulator of early natural killer (NK) cell development and metabolic homeostasis. By employing a conditional knockout model that ablates *Mettl14* from the NK progenitor (NKP) stage, we demonstrate that m^6^A methylation safeguards NK cell ontogeny through two principal mechanisms: ensuring the fidelity of the NKP-to-iNK transcriptional transition and restraining aberrant metabolic and signaling activation to prevent activation-induced cell death (AICD).

Our findings reveal a stage-specific function for METTL14, with its most profound impact exerted during the early commitment and maturation phases. This is evidenced by the severe developmental blockade and lymphopenia in *Mettl14*^CD122-Cre^ mice, contrasting sharply with the minimal phenotypic consequences of Ncr1-Cre-mediated deletion of *Mettl14* in mature NK cells. This temporal requirement is further underscored by the peak expression of m^6^A machinery components in NKPs and the massive dysregulation of m^6^A-modified transcripts during the NKP-to-iNK transition. The transcriptional landscape of *Mettl14*-deficient NKPs was characterized by a premature and disorganized execution of the differentiation program, with key transcription factors like *Nfil3*, *Tbx21*, and *Eomes* being aberrantly expressed. This suggests that m^6^A methylation serves as a crucial timer, ensuring the sequential and coordinated activation of gene networks that guide early lineage progression.

A central and paradoxical phenotype in *Mettl14*^CD122-Cre^ mice was the coexistence of NK cell lymphopenia with a hyperproliferative and hypermetabolic state in the residual iNK and mNK populations. This was driven by the hyperactivation of the mTORC1 signaling axis, likely due to the loss of the negative regulator TSC2, and was accompanied by increased nutrient uptake and mitochondrial biogenesis. However, this heightened metabolic and proliferative activity was unsustainable, leading to genomic stress, as indicated by mitochondrial ROS accumulation and DNA damage response pathway activation. Consequently, it triggered a p53-dependent apoptotic program, which was amplified by the concurrent upregulation of both intrinsic (e.g., BCL-2 family) and extrinsic (e.g., FAS/FASL, TRAIL/DR5) death pathways. This confluence of hyperactivation and apoptosis exemplifies a classic AICD scenario, ultimately accounting for the specific loss of iNK and mNK cells.

The spontaneous hyperactivation of *Mettl14*-deficient NK cells, characterized by elevated CD69/CD25 expression and enhanced cytokine production at steady state, points to a breakdown in signaling quiescence. We identified a key mechanism underlying this phenomenon: the m^6^A-mediated suppression of *Socs3*. The downregulation of SOCS3 protein in knockout cells led to heightened IL-15/JAK-STAT5 signaling, a master pathway for NK cell homeostasis and metabolism. This, combined with concurrent hyperactivation of NF-κB and p38/JNK pathways, creates a signaling environment that primes NK cells for effector responses even in the absence of infection or transformation. Notably, this hyperactive state, while detrimental to homeostasis, translated into superior control of melanoma metastasis in vivo, highlighting a potential trade-off between steady-state population maintenance and acute effector potential.

Although *Mettl14*-deficient NK cells in *Mettl14*^CD122-Cre^ mice exhibited superior antitumor responses, this effect was model-specific. Deletion of *Mettl14* in mature NK cells using Ncr1-Cre did not reproduce the same benefit. Reanalysis of published data by Song et al. showed that *METTL14* expression is significantly downregulated (~49%) in tumor-infiltrating NK cells from hepatocellular carcinoma (HCC) patients relative to non-tumor controls [25]. This finding further suggests that METTL14 downregulation in human tumor-infiltrating NK cells is not consistently associated with increased antitumor function, underscoring the context-dependent nature of its role. Thus, METTL14 serves as a key regulator of both NK cell homeostasis and functional output. Moreover, given its ubiquitous expression, METTL14 is unlikely to be a viable therapeutic target for NK cell-based immunotherapies.

Our research clarifies the non-redundant role of METTL14 compared to its catalytic partner METTL3. The severe phenotype observed in *Mettl14*^CD122-Cre^ mice, which was absent in *Mettl3*^Ncr1*-*Cre^ models from prior studies, suggests METTL14 possesses unique functions beyond its role in the canonical methyltransferase complex. These may include METTL3/m^6^A-independent roles in chromatin regulation or a unique specificity for transcripts that are critical for early lymphoid development.

In conclusion, our work delineates a critical role for METTL14-mediated m^6^A methylation in calibrating the metabolic and signaling fitness of developing NK cells. It acts as a dual-purpose safeguard: first, by enforcing the precise transcriptional timing required for early development, and second, by erecting post-transcriptional checkpoints that prevent metabolic over-activation and AICD, thereby ensuring the generation of a stable and functional NK cell pool.

## Materials and methods

### Animals

CD45.1^+^ congenic mice and *β2* *m*^−/−^ mice were obtained from The Jackson Laboratory (Bar Harbor, Maine, USA). *Rag1*^−/−^*γc*^−/Y^ mice have been described previously [7]. *Mettl14*^fl/fl^ mice were generously provided by Dr. Meng Xu (Tsinghua University, Beijing, China). *Mettl14*^CD122-Cre^ and *Mettl14*^Ncr1-Cre^ mice were generated by crossing *Mettl14*^fl/fl^ mice with CD122-Cre or Ncr1-Cre mice produced in our laboratory [7, 33]. All mice were on the C57BL/6 background and housed in specific pathogen-free facilities at Anhui Medical University (Hefei, China) or Tsinghua University (Beijing, China). Sex- and age-matched littermate mice (6–12 weeks old) were sacrificed for experiments, while 16-week-old *Mettl14*^fl/fl^ and *Mettl14*^CD122-Cre^ mice were employed for long-term monitoring of autoimmune phenotypes. Sample sizes were determined based on prior experience and common practices in the field, and were deemed sufficient to detect biologically meaningful and reproducible effects. The number of animals was minimized in accordance with the reduction principle, while still ensuring reliable results and statistical validity. No blinding was performed for data collection, or data analysis. No mice were excluded from the study, and no randomization was used to assign animals to experimental groups. The animal experiments in this study were complied with ARRIVE guidelines and performed in accordance with protocols approved by the by the Animal Ethics Committee of Anhui Medical University (Approval Number: LLSC20230332).

### Cell lines

The RMA-S cell line (an MHC-I-deficient mouse lymphoma) was originally developed by Klas Kärre at Karolinska Institute (Sweden). YAC-1 and B16-F10 cells were obtained from the American Type Culture Collection (ATCC). All cell lines were cultured in RPMI 1640 medium or DMEM medium (WISENT, QC, Canada), supplemented with 10% heat-inactivated fetal bovine serum and 100 IU/mL penicillin/streptomycin, and maintained at 37 °C in a humidified 5% CO₂ atmosphere. Cells were routinely tested for mycoplasma contamination.

### Flow cytometry and antibodies

Antibodies against the following markers were used in this study: CD3-PE-Cyanine7 (25-0031-82) or PE-eFluor 610 (61-0031-82) or PerCP-Cyanine5.5 (45-0031-82), NK1.1-APC (17-5941-82), CD122-PerCP-eFluor 710 (46-1222-82) or PE (12-1222-82), CD27-PE-Cyanine7 (25-0271-82), CD11b-eFluor 450 (48-0112-82), CD117-APC (17-1171-82), CD127-PE-Cyanine7 (25-1271-82) or PerCP-eFluor 710 (46-1271-82), NKG2D-PE-Cyanine7 (25-5882-82), DNAM-1-APC (17-2261-82), Ly49H-PE-Cyanine7 (25-5886-82), Ly49G.2-FITC (11-5781-82), Ly49A-PE (12-5856-82), Ly49I-PE (12-5895-82), NKG2AB6-PE (12-5897-81), KLRG1-PE-Cyanine5.5 (35-5893-82), CD49b-FITC (11-5971-82), CD45.1-FITC (11-0453-82), CD45.2-PE (12-0454-82), CD25-PE (12-0251-82), CD69-PE (12-0691-82), Ki67-PE (12-5698-82) or PerCP-eFluor 710 (46-5698-82), T-bet- PE (12-5825-82), Eomes-PE-eFluor 610 (61-4877-42), E4BP4-PE (12-5927-82), Bcl2-eFluor 450 (48-6992-42), ID2-PE (12-9475-82), TRAIL-PE (12-5951-82), FASL-PE (12-5911-82), DR5-PE (12-5883-82), CD107a-eFluor 660 (50-1071-82) or PerCP-eFluor 710 (46-1071-82), IFN-γ-PE (12-7311-41) or eFluor 450 (48-7311-82) or PE-Cyanine7 (25-7311-82), TNF-α-PE (12-7321-82), Perforin-PE (12-9392-82), Granzyme B-PE-Cyanine7 (25-8898-82), Flt3-PerCP-eFluor 710 (46-1351-82), CD244.1-APC (17-2441-82), CD19-PE-Cyanine7 (25-0193-82), CD71-FITC (11-0719-42), CD98-PE (12-0981-81), anti-CD16/32 (MA5-51919), Anti-phospho-S6-PE (12-9007-42), anti-Rabbit IgG (H + L) (A-11008), isotype controls (11-4614-80, 12-4888-81, 12-4714-42, 11-4714-81, 12-4321-42), mouse hematopoietic lineage antibody cocktail (13-0032-82, 36-0452-85, 36-0112-85, 13-5921-82, 13-5931-82), Streptavidin-APC-eFluor 780 (47-4317-82), Alexa Fluor™ 488 Annexin V/ Dead Cell Apoptosis Kit (V13242), Vybrant FAM Caspase-3 and -7 Assay Kit (V35118) were purchased from Thermo Fisher Scientific (San Diego, CA, USA). CD49a (142608) and Ly49D (138303) were purchased from BioLegend (San Diego, CA, USA). FAS (561979) was obtained from BD Biosciences (Mississauga, ON Canada). Antibodies against Bim (2933S), p53 (2015), phospho-Akt (Thr308) (13038), phospho-Akt (Ser473) (4060), phospho-Stat5 (Tyr694) (9314), phospho-NF-κB p65 (Ser536) (3033), phospho-p38 MAPK (Thr180/Tyr182) (4511), phospho-PLCγ1 (Tyr783) (14008), phospho-PKC (pan) (gamma Thr514) (38938), phospho-SAPK/JNK (Thr183/Tyr185) (9255), TCF1 (6444), GAPDH (2118) and METTL14 (48699) were obtained from Cell Signaling Technology (Beverly, MA, USA).

To analyze cell surface markers, single-cell suspensions were preincubated with anti-CD16/32 monoclonal antibody to block Fc receptors and then stained with the indicated surface markers. For intracellular or nuclear protein detection, cells were first surface stained, followed by fixation and permeabilization using Fixation/Permeabilization Buffer (BD Biosciences) or Foxp3/Transcription Factor Staining Buffer Set (Thermo Fisher Scientific). Cells were then stained intracellularly with specific antibodies. Caspase-positive cells were detected using DEVD-FMK (Thermo Fisher Scientific) according to the manufacturer’s instructions. All samples were acquired on an LSR Fortessa™ or FACSymphony™ A5 flow cytometer (BD Biosciences) and analyzed using FlowJo software (Version 10.9; BD Biosciences).

### Quantitative PCR

Total RNA was isolated using TRIzol™ Reagent (Takara Bio, Kusatsu, Japan) and used for cDNA synthesis with the HiScript II 1st Strand cDNA Synthesis Kit (Vazyme, Nanjing, China; #R211-01). Quantitative PCR (qPCR) was performed using ChamQ Universal SYBR qPCR Master Mix (Vazyme, #Q311-02) on a CFX Opus™ Real-Time PCR System (Bio-Rad, Hercules, CA, USA) with gene-specific primers (Supplementary Table S1). Fold changes in mRNA expression were calculated using the ΔΔCT method, with *Actb* as the endogenous control. Results are expressed as fold change relative to controls.

### meRIP-seq and data analysis

For meRIP-seq, splenic NK cells (CD3⁻NK1.1⁺) were sorted from wild-type C57BL/6 mice using a BD FACSAria™ III flow cytometer. Total RNA was isolated from ~5 × 10⁷ NK cells and poly(A)^+^ RNA was purified using the Dynabeads™ mRNA Purification Kit (Thermo Fisher Scientific). Purified mRNA (100–150 ng) was fragmented into 100–200-nucleotide fragments using RNA Fragmentation Reagents (Thermo Fisher Scientific). Five percent of the fragmented RNA was reserved as input, while the remainder was subjected to m⁶A enrichment using the EpiMark™ N⁶-Methyladenosine Enrichment Kit (NEB, Ipswich, MA, USA ; #E1610S). Enriched RNA was eluted in 500 µL TRIzol™ and concentrated using the RNA Clean & Concentrator™-5 kit (Zymo Research, Irvine, CA, USA). Indexed cDNA libraries were prepared from both input and m⁶A-IP samples using the SMARTer® Stranded Total RNA-Seq Kit v2 (Takara Bio) for Illumina sequencing. After adapter trimming and quality filtering, sequencing reads were aligned to the mouse genome (mm10 assembly) using HISAT2. m⁶A peaks enriched in IP versus input samples were called using MACS2 and annotated using the ChIPseeker R package. Motif analysis was performed using HOMER. KEGG pathway enrichment was analyzed using the MetaScape online platform.

### RNA-seq and data processing

For RNA-seq analysis of NK cell subsets, splenic NKP (CD3^−^CD122^+^NK1.1^−^CD11b^−^), iNK (CD3^−^CD122^+^NK1.1^+^ CD11b^−^), and mNK (CD3^−^CD122^+^NK1.1^+^CD11b^+^) cells were isolated from both *Mettl14*^WT^ and *Mettl14*^CD122-Cre^ mice using a BD FACSAria™ III flow cytometer. Total RNA was extracted from all sorted cells using the miRNeasy Mini Kit (QIAGEN). Subsequently, RNA libraries were constructed using the SMARTer Stranded Total RNA-Seq Kit (Takara Bio) according to the manufacturer’s protocol. Constructed libraries were sequenced on an Illumina HiSeq X Ten platform. RNA-seq reads were trimmed to remove Illumina adapter sequences using Trim Galore. Ribosomal RNA was then depleted using Bowtie2. The remaining reads were aligned to the mouse genome (mm10) with HISAT2. After removing low-quality reads using SAMtools, uniquely mapped reads were selected and quantified at the gene level using featureCounts. Differential expression analysis was performed using the DESeq2 R package (Bioconductor) to identify differentially expressed genes between sample groups. Enriched KEGG pathways were identified using the online bioinformatics platform MetaScape.

### Generation of mixed bone marrow chimeras

To generate competitive bone marrow chimeras, bone marrow (BM) cells from 5-fluorouracil (5-FU)-treated WT (CD45.1⁺) mice and *Mettl14*^CD122-Cre^ (CD45.2⁺) mice were mixed at a 1:1 ratio and intravenously (i.v.) transferred into lethally irradiated *Rag1*^−/−^*γc*^−/Y^ (CD45.2⁺) mice. Chimeric recipients were assessed by flow cytometry six weeks post-transplantation.

### Ex vivo NK cell functional assay

NK cell function (cytokine production and degranulation) was assessed using splenocytes from naïve mice or lung-infiltrating lymphocytes from mice with metastatic melanoma. Cells were co-cultured for 5 h with target cells (RMA-S or YAC-1) at a 1:1 effector-to-target (E: T) ratio or stimulated on anti-NK1.1-coated plates (2 µg/ml). Negative controls received RPMI 1640 medium alone; positive controls were stimulated with PMA (50 ng/ml) plus ionomycin (1 µM). Throughout the incubation, Brefeldin A, monensin, and anti-CD107a antibody (Thermo Fisher Scientific) were present. Cells were then harvested, surface-stained for CD3 and NKp46, followed by fixation and permeabilization using Fixation/Permeabilization Buffer (BD Biosciences). Intracellular IFN-γ, TNF-α, perforin, and granzyme B were detected using fluorochrome-conjugated antibodies.

### In vivo NK cell-mediated rejection assay

To assess NK cell-mediated rejection in vivo, splenocytes from WT and *β2m*^−/−^ mice were labeled with CFSE at 0.5 mM and 5 mM, respectively. Equal numbers (1 × 10^6^) of labeled cells were co-injected intravenously into recipient mice pre-treated 18 h earlier with 200 μg Poly(I:C) (intraperitoneal). Splenic and lymph node cells were harvested 6 h post-transfer, and the proportion of CFSE^+^ cells was analyzed by flow cytometry. Rejection was quantified as the ratio of *β2m*^−/−^ splenocytes remaining relative to co-injected WT splenocytes.

### Metastatic melanoma model

Mice were intravenously (i.v.) injected with 2 × 10^5^ B16-F10 cells. After 14 days, lungs were harvested and metastatic nodules counted macroscopically. To confirm NK cell involvement, mice were depleted of NK cells via intraperitoneal (i.p.) injection of anti-NK1.1 antibody (clone PK136; 200 µg) on days 4 and day 2 prior to tumor inoculation and then every 3 days post-inoculation.

### Measurement of mitochondrial activity

Splenic cells were suspended in RPMI-1640 medium supplemented with specific fluorescent dyes: 1 μM ER Tracker (Beyotime, Shanghai, China), 75 nM Mitotracker Green (Beyotime), 20 nM TMRE (Beyotime), 10 μM DCFH-DA (Thermo Fisher Scientific), or 1 mM MitoSOX (MitoSOX). The cells were incubated with the respective dyes for 20–30 min at 37 °C in darkness. After washing three times with FBS-free RPMI-1640 medium, surface markers were stained for flow cytometric analysis.

### Assessment of glucose uptake

Freshly isolated splenocytes were suspended in glucose-free RPMI-1640 medium containing 50 μM 2-NBDG (Thermo Fisher Scientific) and incubated for 30 min at 37 °C. Following incubation, cells were stained for surface markers and analyzed by flow cytometry.

### Western blot

Splenic NK cells (CD3⁻NK1.1⁺) were sorted from *Mettl14*^WT^ and *Mettl14*^Ncr1-Cre^ mice using a BD FACSAria™ III flow cytometer. Protein samples from the lysed NK cells were separated by SDS-PAGE and transferred onto polyvinylidene difluoride (PVDF) membranes (Millipore) via wet transfer (Bio-Rad, USA). Membranes were blocked with 5% non-fat milk and incubated overnight at 4 °C with the GAPDH-specific antibody (CST, 2118) at a 1:1000 dilution, followed by washing and incubation with appropriate horseradish peroxidase (HRP)-conjugated secondary antibody. Protein signals were detected using enhanced chemiluminescence (ECL) reagents (Bio-Rad, USA), and visualized using Amersham ImageQuant software (Cytiva, USA). To detect METTL14, membrane stripping was performed to remove antibodies bound to GAPDH, followed by reprobing with the METTL14-specific antibody (CST, 48699).

### Statistical analysis

Data are presented as means ± SEM. Statistical analyses were performed using GraphPad Prism (version 10.0). For comparisons between two independent groups, two-tailed unpaired Student’s *t* tests were used; the *F* test was applied to assess variance equality between the groups. When comparing three or more groups or multiple levels of a single factor, one-way analysis of variance (ANOVA) was conducted, with the Brown–Forsythe test used to evaluate homogeneity of variances. Data were checked for conformity with the assumptions of the statistical tests employed. A *p*-value < 0.05 was considered statistically significant. Significance is denoted as follows: *p < 0.05, **p < 0.01, ***p < 0.001, ****p < 0.0001. Only statistically significant comparisons relative to controls are displayed.

## Supplementary information

SI FIG TAB GEL

## Data Availability

All raw data, source code, and other materials supporting the findings of this study are available from the corresponding author upon reasonable request.

## References

[1] Trapani JA, Smyth MJ. Functional significance of the perforin/granzyme cell death pathway. Nat Rev Immunol. 2002;2:735–747. 10.1038/nri911 12360212

[2] Prager I, Liesche C, van Ooijen H, Urlaub D, Verron Q, Sandström N, et al. NK cells switch from granzyme B to death receptor–mediated cytotoxicity during serial killing. J Exp Med. 2019;216:2113–27. 10.1084/jem.20181454 PMC6719417 31270246

[3] Vivier E, Raulet DH, Moretta A, Caligiuri MA, Zitvogel L, Lanier LL, et al. Innate or Adaptive Immunity? The Example of Natural Killer Cells. Science. 2011;331:44–49. 10.1126/science.1198687 PMC3089969 21212348

[4] Yu J, Freud AG, Caligiuri MA. Location and cellular stages of natural killer cell development. Trends Immunol. 2013;34:573–82. 10.1016/j.it.2013.07.005 PMC3852183 24055329

[5] Marçais A, Cherfils-Vicini J, Viant C, Degouve S, Viel S, Fenis A, et al. The metabolic checkpoint kinase mTOR is essential for IL-15 signaling during the development and activation of NK cells. Nat Immunol. 2014;15:749–57. 10.1038/ni.2936 PMC4110708 24973821

[6] Ma S, Caligiuri MA, Yu J. Harnessing IL-15 signaling to potentiate NK cell-mediated cancer immunotherapy. Trends Immunol. 2022;43:833–47. 10.1016/j.it.2022.08.004 PMC9612852 36058806

[7] Yang MX, Li D, Chang Z, Yang ZZ, Tian ZG, Dong ZJ. PDK1 orchestrates early NK cell development through induction of E4BP4 expression and maintenance of IL-15 responsiveness. J Exp Med. 2015;212:253–65. 10.1084/jem.20141703 PMC4322053 25624444

[8] Wang X, Zhao X-Y. Transcription factors associated with IL-15 cytokine signaling during NK cell development. Front Immunol. 2021;12:610789. 10.3389/fimmu.2021.610789 PMC8013977 33815365

[9] Ramirez K, Chandler KatherineJ, Spaulding C, Zandi S, Sigvardsson M, Graves BarbaraJ, et al. Gene deregulation and chronic activation in natural killer cells deficient in the transcription factor ETS1. Immunity. 2012;36:921–32. 10.1016/j.immuni.2012.04.006 PMC3389314 22608498

[10] Liu J, Wang Z, Hao S, Wang F, Yao Y, Zhang Y, et al. Tcf1 sustains the expression of multiple regulators in promoting early natural killer cell development. Front Immunol. 2021;12:791220. 10.3389/fimmu.2021.791220 PMC8669559 34917097

[11] Gordon ScottM, Chaix J, Rupp LeviJ, Wu J, Madera S, Sun JosephC, et al. The transcription factors T-bet and eomes control key checkpoints of natural killer cell maturation. Immunity. 2012;36:55–67. 10.1016/j.immuni.2011.11.016 PMC3381976 22261438

[12] Aliahmad P, de la Torre B, Kaye J. Shared dependence on the DNA-binding factor TOX for the development of lymphoid tissue–inducer cell and NK cell lineages. Nat Immunol. 2010;11:945–52. 10.1038/ni.1930 PMC2943551 20818394

[13] Fehniger TA, Cai SF, Cao X, Bredemeyer AJ, Presti RM, French AnthonyR, et al. Acquisition of murine NK cell cytotoxicity requires the translation of a pre-existing pool of granzyme B and perforin mRNAs. Immunity. 2007;26:798–811. 10.1016/j.immuni.2007.04.010 17540585

[14] Stetson DB, Mohrs M, Reinhardt RL, Baron JL, Wang Z-E, Gapin L, et al. Constitutive cytokine mRNAs mark natural killer (NK) and NK T cells poised for rapid effector function. J Exp Med. 2003;198:1069–76. 10.1084/jem.20030630 PMC2194220 14530376

[15] Kim T-D, Park JY, Choi I. Post-transcriptional regulation of NK cell activation. Immune Netw. 2009;9:115–121. 10.4110/in.2009.9.4.115 PMC2816943 20157597

[16] Han D, Xu MM. RNA modification in the immune system. Annu Rev Immunol. 2023;41:73–98. 10.1146/annurev-immunol-101921-045401 37126422

[17] Liu J, Yue Y, Han D, Wang X, Fu Y, Zhang L, et al. A METTL3–METTL14 complex mediates mammalian nuclear RNA N6-adenosine methylation. Nat Chem Biol. 2013;10:93–95. 10.1038/nchembio.1432 PMC3911877 24316715

[18] Zaccara S, Ries RJ, Jaffrey SR. Reading, writing and erasing mRNA methylation. Nat Rev Mol Cell Biol. 2019;20:608–24. 10.1038/s41580-019-0168-5 31520073

[19] Lee H, Bao S, Qian Y, Geula S, Leslie J, Zhang C, et al. Stage-specific requirement for Mettl3-dependent m6A mRNA methylation during haematopoietic stem cell differentiation. Nat Cell Biol. 2019;21:700–9. 10.1038/s41556-019-0318-1 PMC6556891 31061465

[20] Dong L, Chen C, Zhang Y, Guo P, Wang Z, Li J, et al. The loss of RNA N6-adenosine methyltransferase Mettl14 in tumor-associated macrophages promotes CD8+ T cell dysfunction and tumor growth. Cancer Cell. 2021;39:945–.e910. 10.1016/j.ccell.2021.04.016 34019807

[21] Li H-B, Tong J, Zhu S, Batista PJ, Duffy EE, Zhao J, et al. m6A mRNA methylation controls T cell homeostasis by targeting the IL-7/STAT5/SOCS pathways. Nature. 2017;548:338–42. 10.1038/nature23450 PMC5729908 28792938

[22] Tong J, Cao G, Zhang T, Sefik E, Amezcua Vesely MC, Broughton JP, et al. m6A mRNA methylation sustains Treg suppressive functions. Cell Res. 2018;28:253–6. 10.1038/cr.2018.7 PMC5799823 29303144

[23] Yao Y, Yang Y, Guo W, Xu L, You M, Zhang Y-C, et al. METTL3-dependent m6A modification programs T follicular helper cell differentiation. Nat Commun. 2021;12:1333. 10.1038/s41467-021-21594-6 PMC7910450 33637761

[24] Xiao Z, Wang S, Tian Y, Lv W, Sheng H, Zhan M, et al. METTL3-mediated m6A methylation orchestrates mRNA stability and dsRNA contents to equilibrate γδ T1 and γδ T17 cells. Cell Rep. 2023;42:112684. 10.1016/j.celrep.2023.112684 37355989

[25] Song H, Song J, Cheng M, Zheng M, Wang T, Tian S, et al. METTL3-mediated m6A RNA methylation promotes the anti-tumour immunity of natural killer cells. Nat Commun. 2021;12:5522. 10.1038/s41467-021-25803-0 PMC8448775 34535671

[26] Wang H, Hu X, Huang M, Liu J, Gu Y, Ma L, et al. Mettl3-mediated mRNA m6A methylation promotes dendritic cell activation. Nat Commun. 2019;10:1898. 10.1038/s41467-019-09903-6 PMC6478715 31015515

[27] Cao L, Morgun E, Genardi S, Visvabharathy L, Cui Y, Huang H, et al. METTL14-dependent m6A modification controls iNKT cell development and function. Cell Rep. 2022;40:111156. 10.1016/j.celrep.2022.111156 PMC9495716 35926466

[28] Vu LP, Pickering BF, Cheng Y, Zaccara S, Nguyen D, Minuesa G, et al. The N6-methyladenosine (m6A)-forming enzyme METTL3 controls myeloid differentiation of normal hematopoietic and leukemia cells. Nat Med. 2017;23:1369–76. 10.1038/nm.4416 PMC5677536 28920958

[29] Han D, Liu J, Chen C, Dong L, Liu Y, Chang R, et al. Anti-tumour immunity controlled through mRNA m6A methylation and YTHDF1 in dendritic cells. Nature. 2019;566:270–4. 10.1038/s41586-019-0916-x PMC6522227 30728504

[30] Zheng Z, Zhang L, Cui X-L, Yu X, Hsu PJ, Lyu R, et al. Control of early B cell development by the RNA N6-methyladenosine methylation. Cell Rep. 2020;31. 10.1016/j.celrep.2020.107819 PMC7371152 32610122

[31] Kim SM, Oh SC, Lee SY, Kong LZ, Lee JH, Kim TD. FTO negatively regulates the cytotoxic activity of natural killer cells. EMBO Rep. 2023;24. 10.15252/embr.202255681 PMC10074099 36744362

[32] Ma S, Yan J, Barr T, Zhang J, Chen Z, Wang L-S, et al. The RNA m6A reader YTHDF2 controls NK cell antitumor and antiviral immunity. J Exp Med. 2021;218:e20210279. 10.1084/jem.20210279 PMC8225680 34160549

[33] Chen SS, Li ZH, Feng J, Quan YH, He JM, Hao JQ, et al. Dual activity of type III PI3K kinase Vps34 is critical for NK cell development and senescence. Adv Sci. 2024;11:e2309315. 10.1002/advs.202309315 PMC11151045 38544346

[34] Chiossone L, Chaix J, Fuseri N, Roth C, Vivier E, Walzer T. Maturation of mouse NK cells is a 4-stage developmental program. Blood. 2009;113:5488–96. 10.1182/blood-2008-10-187179 19234143

[35] Spinelli JB, Haigis MC. The multifaceted contributions of mitochondria to cellular metabolism. Nat Cell Biol. 2018;20:745–54. 10.1038/s41556-018-0124-1 PMC6541229 29950572

[36] Morita M, Gravel S-P, Chénard V, Sikström K, Zheng L, Alain T, et al. mTORC1 controls mitochondrial activity and biogenesis through 4E-BP-dependent translational regulation. Cell Metab. 2013;18:698–711. 10.1016/j.cmet.2013.10.001 24206664

[37] Wang XF, Sun R, Hao XL, Lian ZX, Wei HM, Tian ZG. IL-17 constrains natural killer cell activity by restraining IL-15-driven cell maturation via SOCS3. Proc Natl Acad Sci USA. 2019;116:17409–18. 10.1073/pnas.1904125116 PMC6717263 31405974

[38] Khan AU, Ali AK, Marr B, Jo D, Ahmadvand S, Fong-McMaster C, et al. The TNFα/TNFR2 axis mediates natural killer cell proliferation by promoting aerobic glycolysis. Cell Mol Immunol. 2023;20:1140–55. 10.1038/s41423-023-01071-4 PMC10541863 37553427

[39] Almishri W, Santodomingo-Garzon T, Le T, Stack D, Mody CH, Swain MG. TNFα augments cytokine-induced NK cell IFNγ production through TNFR2. J Innate Immun. 2016;8:617–29. 10.1159/000448077 PMC6738838 27560480

[40] Brenner D, Blaser H, Mak TW. Regulation of tumour necrosis factor signalling: live or let die. Nat Rev Immunol. 2015;15:362–74. 10.1038/nri3834 26008591

