# Supplementary material for: METTL14-mediated m6A methylation orchestrates early NK cell development by maintaining metabolic fitness
Source: Cell Death Dis. 2026 Jul 28;17(1):905. doi: 10.1038/s41419-026-09069-2 (PMC13690738; doi:10.1038/s41419-026-09069-2)

For **Cell Death & Disease**

METTL14-mediated m^6A Methylation Orchestrates Early NK Cell Development by Maintaining Metabolic Fitness

Huan Ma[1#], Zhenzhen Tu[1#], Surong Deng[2,1], Yirong Sun[3,1], Shuxin Wang[2,1], Hongxia Wang[3,1], Chen Cheng[1], Xiang Li[1], Zhongjun Dong[1,2,3,4,5,6,7*], Shasha Chen[1,2,3,5,6,7*]

[1] The First Affiliated Hospital of Anhui Medical University and Institute of Clinical Immunology, Anhui Medical University, Hefei, 230032, China.

[2] Department of Immunology, School of Basic Medical Sciences, Anhui Medical University, Hefei, 230032, China.

[3] Department of Immunobiology, College of Life Sciences, Anhui Medical University, Hefei, 230032, China.

[4] School of Basic Medicine and Institute for Immunology, Tsinghua University, Beijing, 100084, China.

[5] Innovative Institute of Tumor Immunity and Medicine (ITIM), Hefei, 230032, China.

[6] Anhui Province Key Laboratory of Tumor Immune Microenvironment and Immunotherapy, Hefei, 230032, China.

[7] Inflammation and Immune Mediated Diseases Laboratory of Anhui Province, Anhui Medical University, Hefei, 230032, China

# These authors contribute equally.

*Correspondence: dongzj@mail.tsinghua.edu.cn; chenshasha2022@fy.ahmu.edu.cn.

SUPPLEMENTAL FIGURE LEGENDS

Figure S1. Quality control analysis of MeRIP-seq data using deepTools and Picard.

A-B, deepTools QC results: **(A)** Spearman correlation of read counts across gene regions; **(B)** Principal component analysis (PCA) of read counts across gene regions. **C-D,** Picard QC result: **(C)** RNA-Seq coverage versus transcript position (All Reads), showing normalized read coverage along the length of transcripts (x-axis: transcript position, 0–100%; y-axis: normalized coverage). **(D)** Distribution of read coverage across the genome or specified regions.

Figure S2. Immune cell frequencies in lymphoid organs of *Mettl14*^{WT} and *Mettl14*$^{CD122\text{-}Cre}$ mice.

A, RT-PCR analysis of *Mettl14* expression in splenic NK cells from the indicated mice (n = 6 mice/group). **B-C,** Representative FCM profiles **(B)**, frequencies, and absolute numbers **(C)** of T cells ($CD3^{+}CD19^{-}$) and B cells ($CD3^{-}CD19^{+}$) in the spleen and BM of the indicated mice (n = 6–15 mice/group). **D-E,** Representative FCM profiles **(D)**, frequencies, and absolute numbers **(E)** of NKT cells ($CD3^{+}NK1.1^{+}$) in the spleen (SP), liver, and thymus (TH) of the indicated mice (n = 7–20 mice/group each. **F-H,** Representative FCM profiles **(F)**, frequencies, and absolute numbers of **(G)** conventional NK cells (cNK) ($CD3^{-}NK1.1^{+}CD49b^{+}$) and **(H)** type 1 innate lymphoid cells (ILC1) ($CD3^{-}NK1.1^{+}CD49a^{+}$) in the liver of the indicated mice (n = 4 mice/group). **I,** Representative FCM profiles (left) and frequencies (right) of DN, CD27 SP, DP, and CD11b SP subsets among the donor-derived splenic NK cells from the competitive chimera mice 8 weeks after BM reconstitution (n = 5 mice/group). Data are pooled from 2–4 independent experiments (C, E) or representative of three independent experiments (G, H, I). Data are presented as means ± SEM. Statistical significance was determined by two-tailed unpaired Student's t-tests. $^{*}P < 0.05$; $^{**}P < 0.01$; $^{***}P < 0.001$; $^{****}P < 0.0001$.

Figure S3. *Mettl14*-deficient NK cells exhibit extensive transcriptional changes.

A-B, Volcano plots illustrating differentially expressed transcripts in iNK **(A)** and mNK **(B)** cells from *Mettl14*$^{CD122\text{-}Cre}$ versus *Mettl14*^{WT} mice (adjusted $P \leq 0.05$; $\log_2$ fold-change ≥ 0.5). **C-D,** GSEA demonstrating significantly enriched pathways in iNK **(C)** and mNK **(D)** cells from *Mettl14*$^{CD122\text{-}Cre}$ versus *Mettl14*^{WT} mice. **E- F,** KEGG pathway enrichment of downregulated DEGs in iNK **(E)** and mNK **(F)** cells from *Mettl14*$^{CD122\text{-}Cre}$ versus *Mettl14*^{WT} mice.

Figure S4. ***Mettl14*$^{\mathbf{CD122\text{-}Cre}}$ mice develop spontaneous autoimmune-like manifestations. A**, Representative morphology of spleens and lymph nodes from 16-week-old *Mettl14*^{WT}and *Mettl14*$^{CD122\text{-}Cre}$mice. **B**, Absolute numbers of splenocytes in 16-week-old *Mettl14*^{WT}and *Mettl14*$^{CD122\text{-}Cre}$mice (n = 5–6 mice/group each). Data are presented as means ± SEM. Statistical significance was determined by two-tailed unpaired Student's t-tests. $^{**}P < 0.01$.

Figure S5. METTL14 is dispensable for late-stage NK cell maturation and function.

A, Western blot analysis of METTL14 protein in sorted NK cells from *Mettl14*^{WT} and *Mettl14*$^{Ncr1\text{-}Cre}$ mice. **B-D,** Representative FCM profiles **(B)**, frequencies **(C)**, and absolute numbers **(D)** of NK cells in the spleen and BM of *Mettl14*^{WT} and *Mettl14*$^{Ncr1\text{-}Cre}$ mice (n = 10–11 mice/group). **E-F,** Representative FCM profiles **(E)** and frequencies **(F)** of DN, CD27 SP, DP, and CD11b SP cell subsets among splenic and BM NK cells of the indicated mice (n = 6–7 mice/group). **G,** Frequencies of NK cells expressing maturation receptors in the spleen of the indicated mice. 3 mice/group each. **H,** PolyI:C-activated splenocytes from *Mettl14*^{WT} and *Mettl14*$^{Ncr1\text{-}Cre}$ mice were either unstimulated (NC) or stimulated with RMA-S, YAC-1, or PMA plus ionomycin. Cumulative results show the percentages of IFN-γ^+, TNF-α^+ and CD107a$^+$ splenic NK cells (n = 3 mice/group). **I,** Representative FCM profiles (left) and cumulative rejection efficiency (right) of splenocytes 6 hours after intravenous injection of a 1:1 mixture of CFSE-labeled WT and $\beta_2m^{-/-}$ splenocytes into indicated mice (n = 5–8 mice/group). **J,** In the B16 metastasis model, indicated mice were intravenously injected with B16-F10 cells (2×10^5). Mice were euthanized 14 days post-inoculation. Representative lung morphology (left) and quantification of total metastatic lung nodules (right) are shown (n = 7–11 mice/group). Data are pooled from 2–3 independent experiments (C, D, F, J) or representative of three independent experiments (G, H, I). Data are presented as means ± SEM. Statistical significance was determined by two-tailed unpaired Student's t-tests.

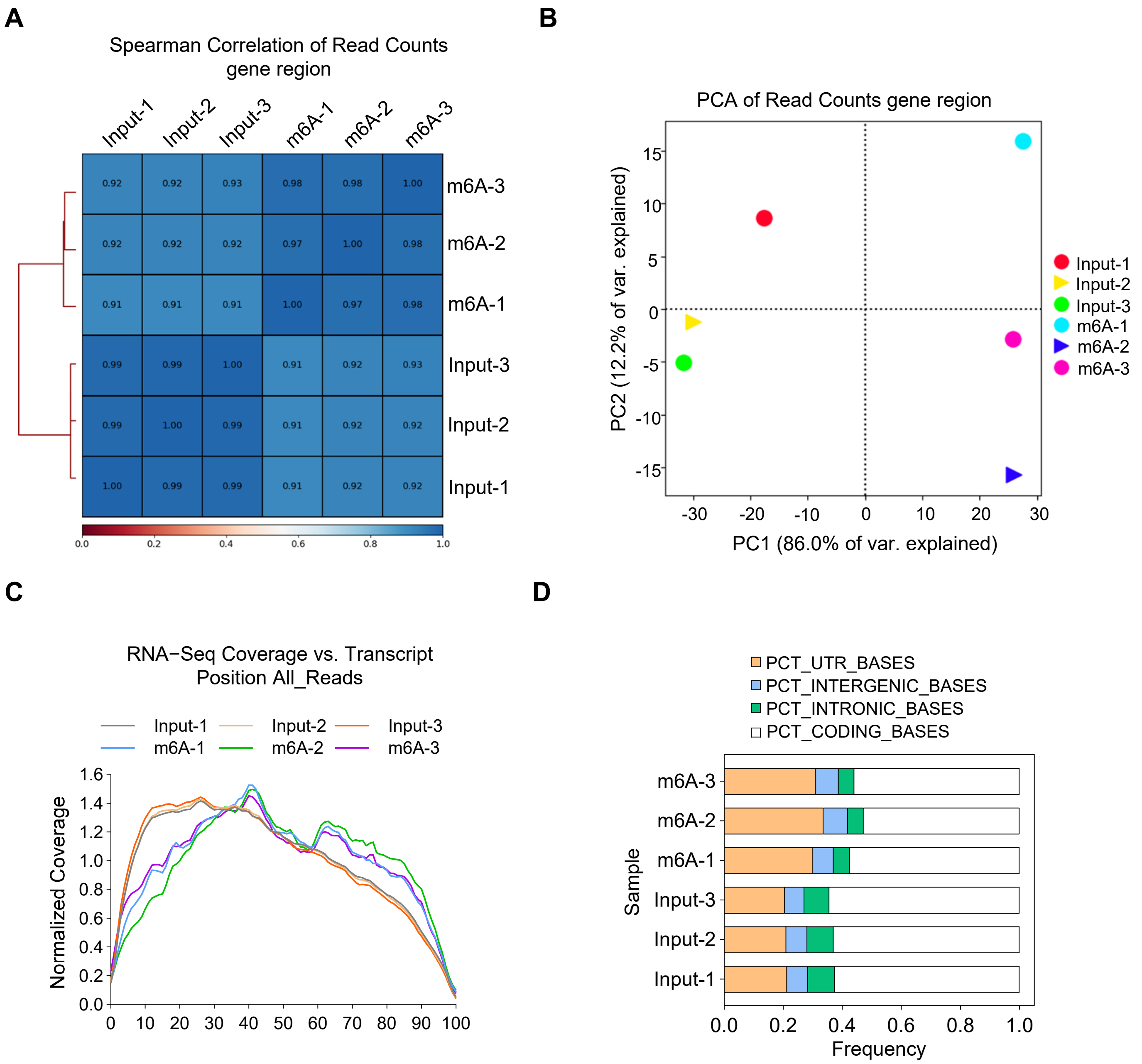

Figure S1. Quality control analysis of MeRIP-seq data using deepTools and Picard

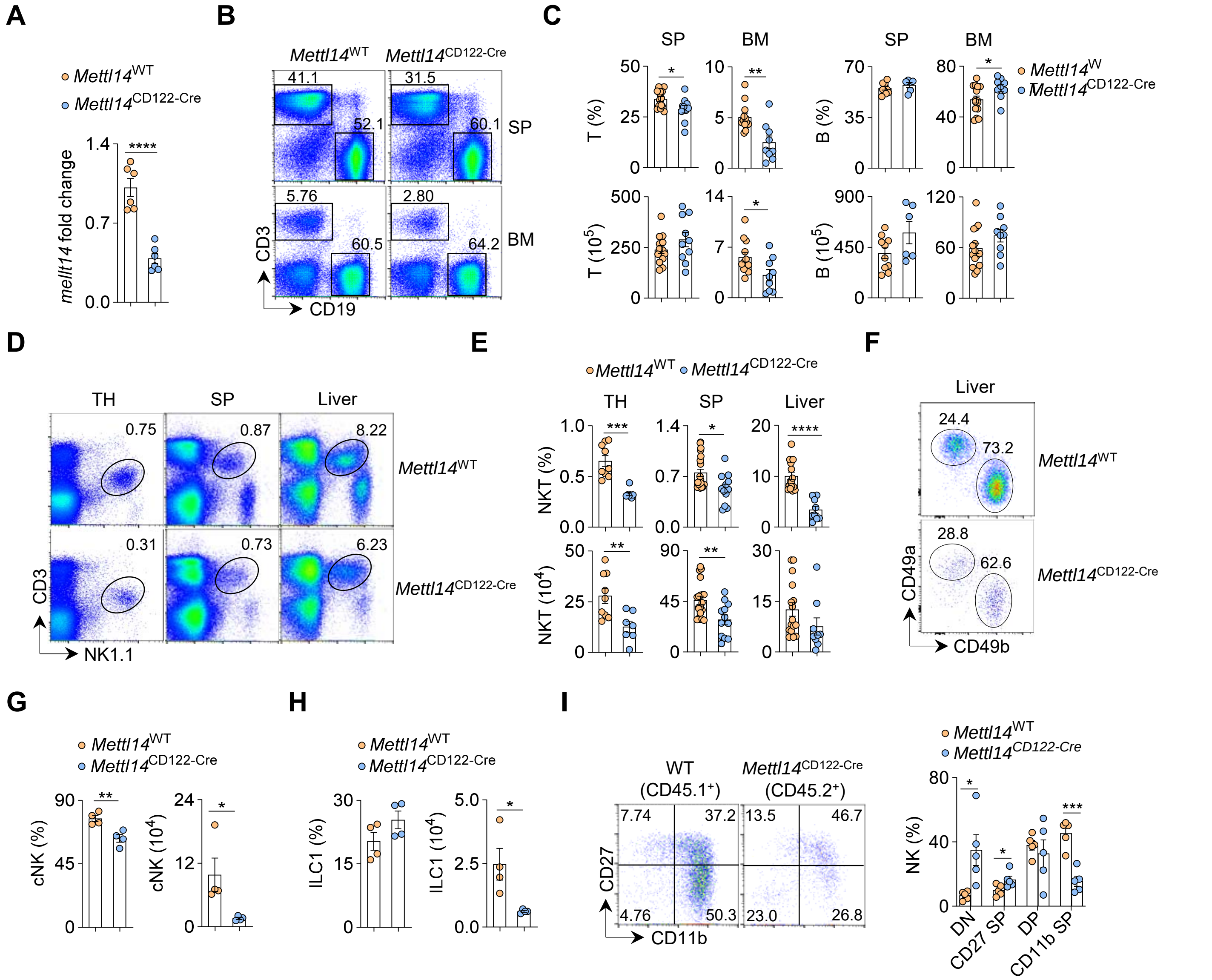

Figure S2. Immune cell frequencies in lymphoid organs of *Mettl14*^{WT} versus *Mettl14*$^{CD122\text{-}Cre}$ mice.

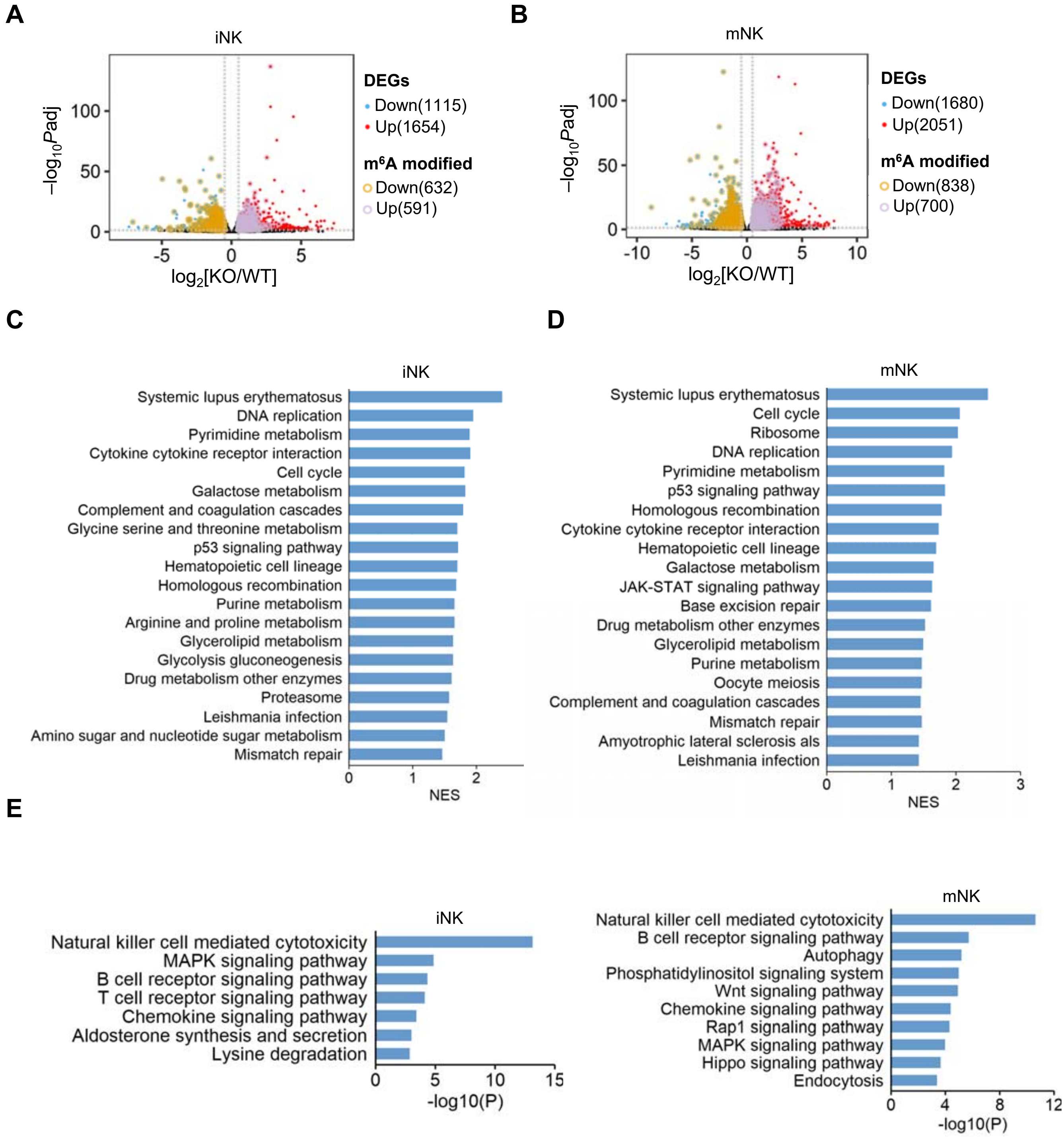

Figure S3. ***Mettl14*-deficient NK cells exhibit extensive transcriptional changes.**

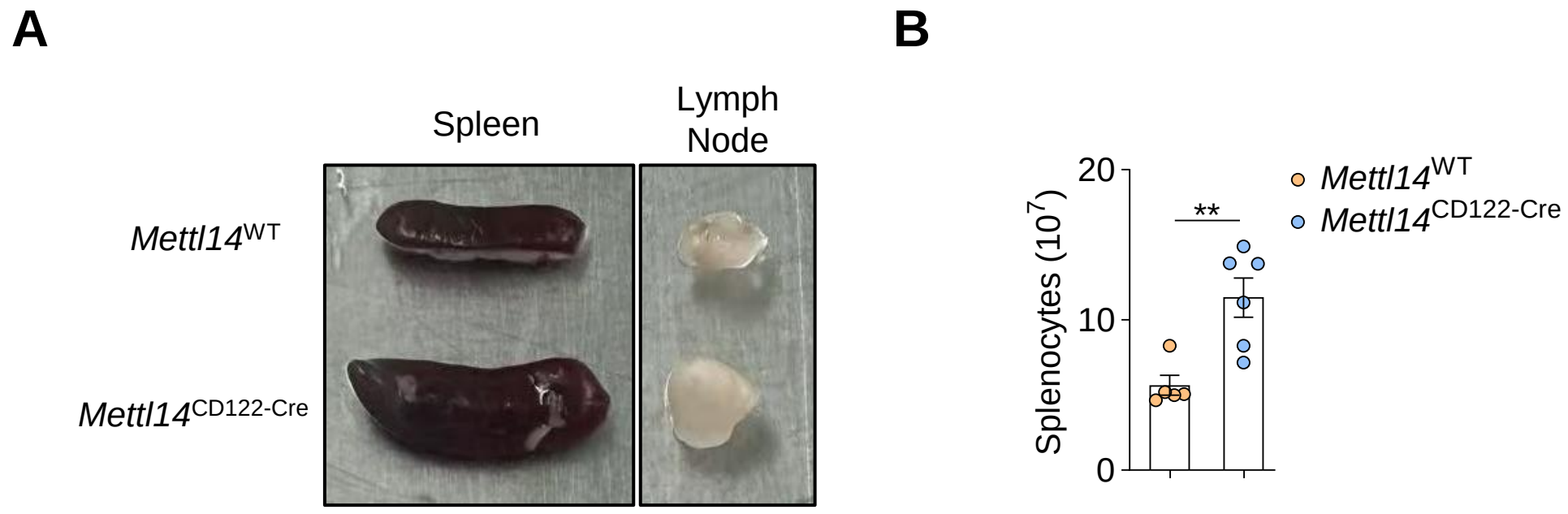

Figure S4. *Mettl14*$^{\text{CD122-Cre}}$ mice develop spontaneous autoimmune-like manifestations.

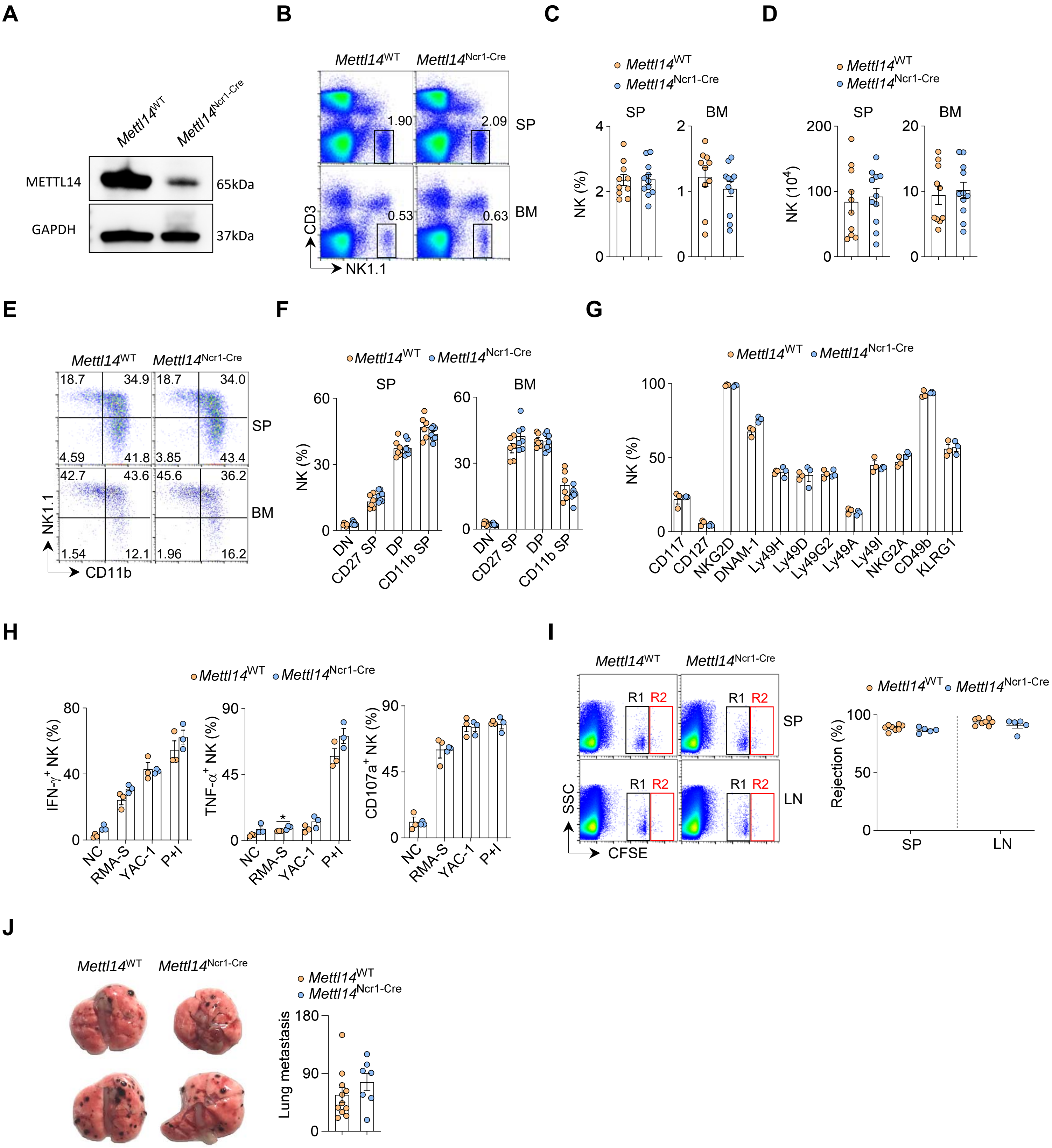

Figure S5. METTL14 is dispensable for late-stage NK cell maturation and function.

Table S1. Primer sequences for Real-Time PCR

Gene	Primer	Species
Actb	GGCTGTATTCCCCTCCATCG (forward)<br>CCAGTTGGTAACAATGCCATGT (reverse)	mouse
Mettl3	AGCAGAGCAAGAGACGAATTATC (forward)<br>GGTGGAAAGAGTCGATCAGCA (reverse)	mouse
Mettl14	CTGAGAGTGCGGATAGCATTG (forward)<br>GAGCAGATGTATCATAGGAAGCC (reverse)	mouse
Ythdf1	GCATCAGAAGGATGCAGTTCATG (forward)<br>GATGGTGGATAGTAACTGGACAG (reverse)	mouse
Ythdf2	GGTTCTGTGCATCAAAAGGATGG (forward)<br>CCAAAGAATAGGAAAAGCCAATGG (reverse)	mouse
Ythdf3	GGTTCGATTCATCAAAAAGATGCTG (forward)<br>GATCTGACATTGGTGGATAGCTG (reverse)	mouse
Ythdc1	CCAAGTCTCCTACACCAGATGG (forward)<br>AGAACCACTGCTTCCTGTCTCG (reverse)	mouse
Ythdc2	TGTGTCTGAAGAGTATGA (forward)<br>CATCTCCTTGATTAACCAT (reverse)	mouse
Alkbh5	CGCGGTCATCAACGACTACC (forward)<br>ATGGGCTTGAACTGGAACTTG (reverse)	mouse
Fto	TTCATGCTGGATGACCTCAATG (forward)<br>GCCAACTGACAGCGTTCTAAG (reverse)	mouse
Cdk1 F1	AGAAGGTACTTACGGTGTGGT (forward)<br>GAGAGATTTCCCGAATTGCAGT (reverse)	mouse
Cdk2 F1	CCTGCTTATCAATGCAGAGGG (forward)<br>GTGCTGGGTACACACTAGGTG (reverse)	mouse
Cdk4 F1	ATGGCTGCCACTCGATATGAA (forward)<br>TCCTCCATTAGGAACTCTCACAC (reverse)	mouse
Cdk6 F1	GGCGTACCCACAGAAACCATA (forward)<br>AGGTAAGGGCCATCTGAAAACT (reverse)	mouse
Ccna2	GCCTTCACCATTCATGTGGAT (forward)<br>TTGCTGCGGGTAAAGAGACAG (reverse)	mouse
Ccnb1	AAGGTGCCTGTGTGTGAACC (forward)<br>GTCAGCCCCATCATCTGCG (reverse)	mouse
Ccnb2	GCCAAGAGCCATGTGACTATC (forward)<br>CAGAGCTGGTACTTTGGTGTTC (reverse)	mouse
Ccne1	GTGGCTCCGACCTTTCAGTC (forward)<br>CACAGTCTTGTCAATCTTGGCA (reverse)	mouse
Ccne2	ATGTCAAGACGCAGCCGTTTA (forward)<br>GCTGATTCCTCCAGACAGTACA (reverse)	mouse
Cdc45	GATTTCCGCAAGGAGTTCTACG (forward)<br>TACTGGACGTGGTCACACTGA (reverse)	mouse
Cdkn1a	CCTGGTGATGTCCGACCTG (forward)<br>CCATGAGCGCATCGCAATC (reverse)	mouse
Tsc2	GCCTCTGTTCATTATCACCC (forward)<br>AGGTTACGCATCAACTTCC (reverse)	mouse
Cish	ATGGTCCTTTGCGTACAGGG (forward)<br>GGAATGCCCCAGTGGGTAAG (reverse)	mouse
Socs3	CCCTTGCAGTTCTAAGTTCAACA (forward)<br>ACCTTTGACAAGCGGACTCTC (reverse)	mouse
Ptpn11	AGAGGGAAGAGCAAATGTGTCA (forward)<br>CTGTGTTTCCTTGTCCGACCT (reverse)	mouse

Uncropped Western blot for Figure S5A.

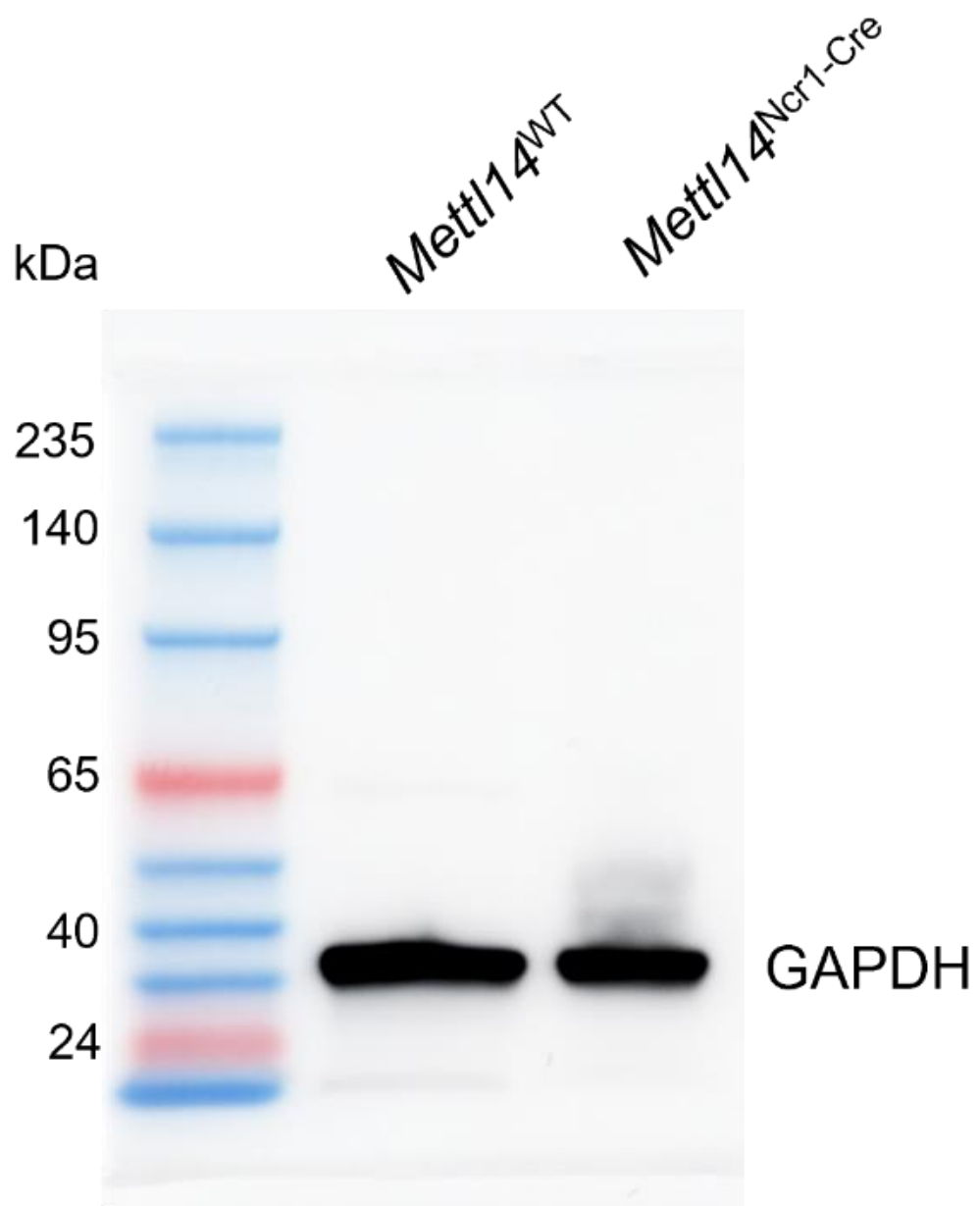

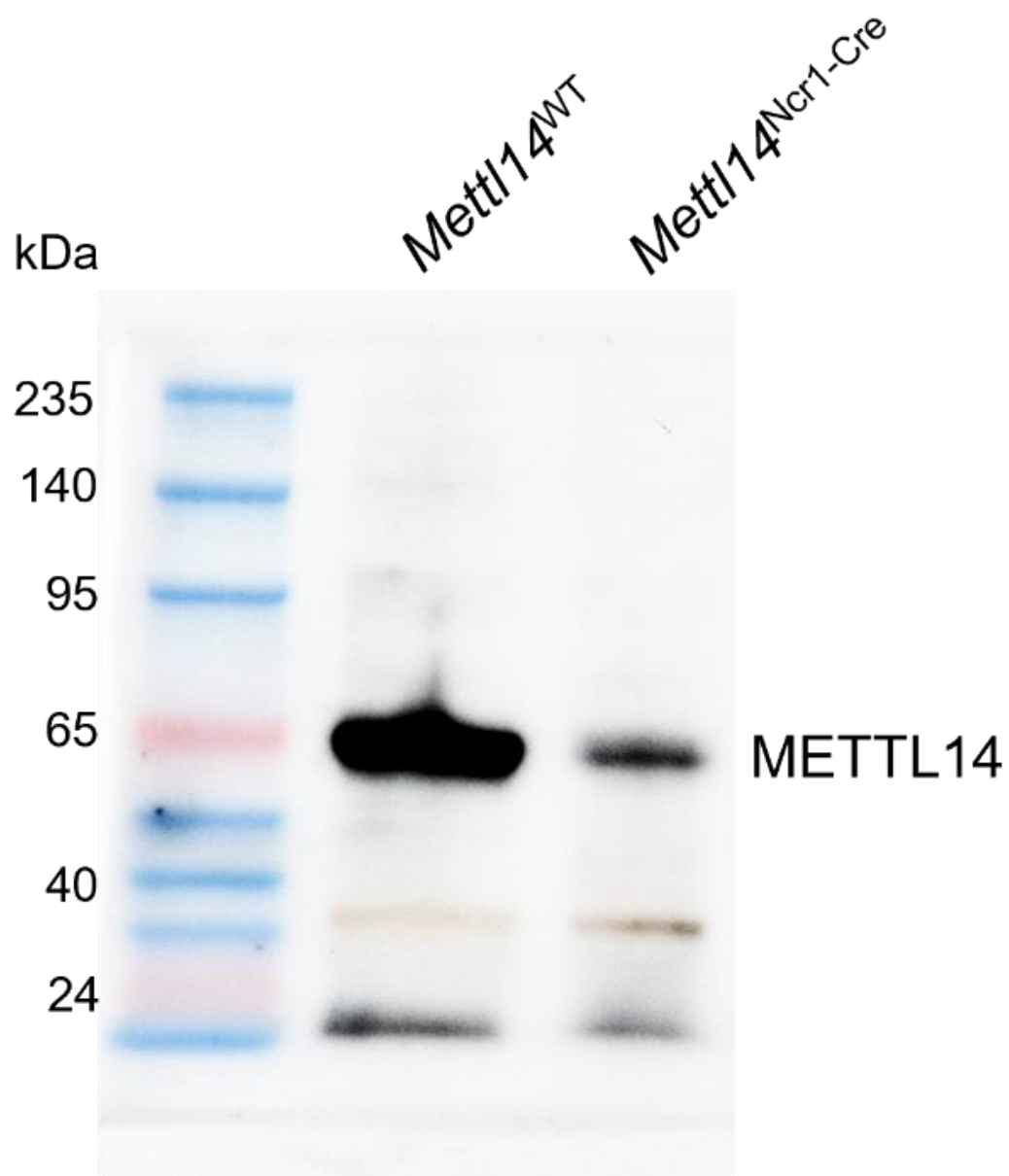

Supplement: Supplementary file 1 [file 41419_2026_9069_MOESM1_ESM.pdf]
